# Association Between Statin Use at the Time of Intra-abdominal Surgery and Postoperative Adhesion-Related Complications and Small-Bowel Obstruction

**DOI:** 10.1001/jamanetworkopen.2020.36315

**Published:** 2021-02-03

**Authors:** Frank I. Scott, Ravy K. Vajravelu, Ronac Mamtani, Nicholas Bianchina, Najjia Mahmoud, Jason K. Hou, Qufei Wu, Xingmei Wang, Kevin Haynes, James D. Lewis

**Affiliations:** 1 Division of Gastroenterology and Hepatology, University of Colorado Anschutz Medical Campus, Aurora; 2 Center for Clinical Epidemiology and Biostatistics, University of Pennsylvania, Philadelphia; 3 Division of Gastroenterology, University of Pennsylvania, Philadelphia; 4 Abramson Cancer Center, University of Pennsylvania, Philadelphia; 5 University of Colorado School of Medicine, Aurora; 6 Department of Surgery, University of Pennsylvania, Philadelphia; 7 Center for Innovations in Quality, Effectiveness and Safety, Michael E. DeBakey Veterans Affairs Medical Center, Houston, Texas; 8 Department of Scientific Affairs, HealthCore Inc, Wilmington, Delaware

## Abstract

**Importance:**

Adhesion-related complications (ARCs), including small-bowel obstruction, are common complications of intra-abdominal surgery. Statins, which have antifibrotic pleiotropic effects, inhibit adhesion formation in murine models but have not been assessed in humans.

**Objective:**

To assess whether statin use at the time of intra-abdominal surgery is associated with a reduction in ARCs.

**Design, Setting, and Participants:**

These 2 separate retrospective cohort studies (The Health Improvement Network [THIN] and Optum’s Clinformatics Data Mart [Optum]) compared adults receiving statins with those not receiving statins at the time of intra-abdominal surgery. Individuals undergoing intra-abdominal surgery from January 1, 1996, to December 31, 2013, in the United Kingdom and from January 1, 2000, to December 31, 2016, in the US were included in the study. Those with obstructive events before surgery or a history of inflammatory bowel disease were excluded. Data analysis was performed from September 1, 2012, to November 24, 2020.

**Exposure:**

The primary exposure was statin use at the time of surgery.

**Main Outcomes and Measures:**

The primary outcome was ARCs, defined as small-bowel obstruction or need for adhesiolysis, occurring after surgery. Sensitivity analyses included statin use preceding but not concurrent with surgery, fibrate use, and angiotensin-converting enzyme inhibitor use. All analyses were adjusted for age, sex, and conditions associated with microvascular disease, such as hypertension, hyperlipidemia, obesity, and tobacco use; surgical approach and site; and diagnosis of a malignant tumor.

**Results:**

A total of 148 601 individuals met the inclusion criteria for THIN (mean [SD] age, 49.6 [17.7] years; 70.1% female) and 1 188 217 for Optum (mean [SD] age, 48.2 [16.4] years; 72.6% female). A total of 2060 participants (1.4%) experienced an ARC in THIN and 54 136 (4.6%) in Optum. Statin use at the time of surgery was associated with decreased risk of ARCs (THIN: adjusted hazard ratio [HR], 0.81; 95% CI, 0.71-0.92; Optum: adjusted HR, 0.92; 95% CI, 0.90-0.95). Similar associations were appreciated between statins and small-bowel obstruction (THIN: adjusted HR, 0.80; 95% CI, 0.70-0.92; Optum: adjusted HR, 0.88; 95% CI, 0.85-0.91).

**Conclusions and Relevance:**

This study’s findings suggest that, among individuals in 2 separate cohorts undergoing intra-abdominal surgery, statin use may be associated with a reduced risk of postoperative ARCs. Statins may represent an inexpensive, well-tolerated pharmacologic option for preventing ARCs.

## Key Points

**Question:**

Are statins associated with a reduced risk of postoperative adhesion-related complications?

**Findings:**

In these 2 separate retrospective cohort studies using population-representative data sets including more than 1.3 million individuals, statin use at the time of intra-abdominal surgery was significantly associated with an 8% to 19% reduction in adhesion-related complications and a 12% to 20% reduction in small-bowel obstruction after intra-abdominal surgery.

**Meaning:**

The findings of this cohort study suggest that statin use at the time of intra-abdominal surgery may be associated with a decreased risk of postoperative adhesion-related complications.

## Teaser

This analysis of 2 large, population-representative cohort studies of patients undergoing intra-abdominal surgery in the UK and US assesses whether statin use at the time of surgery is associated with a reduction in adhesion-related complications.

## Introduction

More than 90% of patients develop adhesions after intra-abdominal surgery.^1,2^ Adhesion-related complications (ARCs) occur in up to 5% of patients undergoing these operations and are responsible for significant morbidity and mortality. More than 70% of small-bowel obstructions (SBOs) and 40% of cases of infertility are secondary to adhesions.^3,4,5^ Future operations in patients with adhesions are associated with inadvertent enterotomy rates of 10% to 20%, resulting in an estimated mortality of 13%.^6^

The pathogenesis of adhesion formation is believed to involve foreign-body exposure, surgical location, and tissue hypoxia. These factors result in inflammation and a profibrotic cytokine milieu.^7,8,9,10,11,12,13^ Given the high prevalence of adhesion-related adverse events, a concerted effort has been made to prevent their formation. Expert-based recommendations have focused on surgical technique and laparoscopy, although the effect of such changes on reducing ARC remains modest.^14,15^ Barrier agents have been developed, but their efficacy in reducing adhesion-related SBOs remains uncertain, and their use is associated with an increased risk of abscess formation.^16,17^ An analysis^18^ of secular trends in ARCs, including SBO and adhesiolysis, during the past 20 years found no significant change in rates of these complications despite these methods and devices.

The key to reducing ARCs may lie in directly inhibiting the profibrotic cytokines that promote their formation. In vitro studies^8,9,10,11,12^ have found that adhesion-related fibroblasts express higher rates of transforming growth factor β, cyclooxygenase 2, and interleukin 10 and reduced levels of tissue plasminogen activator (tPA). Current therapies do not directly target this profibrotic milieu.

β-Hydroxy-β-methylglutaryl-CoA (HMG-CoA) reductase inhibitors (statins) may inhibit profibrotic cytokines. Statins directly influence adhesion-related cytokines in vitro and in murine models through inhibiting farnesyl pyrophosphate and geranylgeranyl pyrophosphate formation. Geranylgeranyl pyrophosphate is a key precursor for RhoA and Rho-associated protein kinase activation.^19^ Rho-associated protein kinase is a regulator of tPA-1 and plasminogen activator inhibitor 1. Through inhibiting farnesyl pyrophosphate, statins downregulate plasminogen activator inhibitor while increasing tPA expression^20,21,22^ (Figure). Statins also directly modulate profibrotic cytokines, including transforming growth factor β.^23,24^

**Figure.**
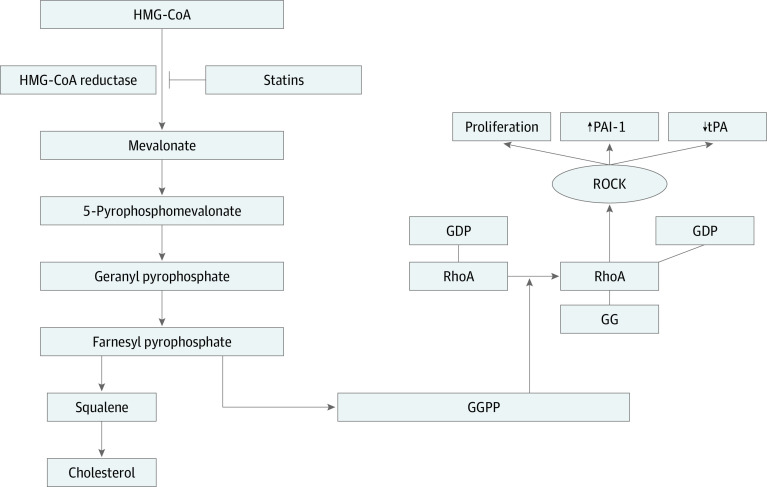
Biochemical Role of Statins in Adhesion Inhibition β-Hydroxy-β-methylglutaryl-CoA (HMG-CoA) reductase inhibitors (statins) inhibit the production of geranylgeranyl (GG) pyrophosphate (GGPP), a key component of lipidation and activation of the small GTPase RhoA. RhoA can subsequently influence Rho-associated protein kinase (ROCK), resulting in the upregulation of plasminogen activator inhibitor 1 (PAI-1) (upward arrow) and downregulation of tissue plasminogen activator (tPA) (downward arrow), both modifications considered to be profibrotic. GDP indicates geranyl diphosphate.

The association of statins with adhesion formation has been confirmed in murine models. Aarons et al^25^ found a significant reduction in adhesion formation in mice undergoing intra-abdominal surgery when exposed to statins intraoperatively. This finding was reversed with coadministration of mevalonate, a downstream HMG-CoA reductase product. These findings were confirmed in rats.^26^ To date, the association of statins with adhesion formation in humans has not been assessed. In addition, no data regarding statins and ARCs, such as SBOs or need for adhesiolysis, are available to our knowledge. In this study, we examined the association between statin use at the time of intra-abdominal surgery and subsequent risks of ARCs in 2 large, population-representative cohorts in the United Kingdom and the US, while adjusting for other comorbidities and surgical factors that could increase the risk of ARCs.

## Methods

### Study Design

Two retrospective cohort studies were performed to assess the association between statin use at the time of intra-abdominal surgery and postoperative ARCs. The first study used data collected prospectively in the scope of routine care from January 1, 1995, to December 31, 2013, within The Health Improvement Network (THIN). THIN includes data derived from the electronic medical records of more than 12 million patients, with 3.6 million active patients within 550 general practices within the United Kingdom.^27^ Mean follow-up time within THIN exceeds 5 years. THIN represents approximately 6% of the UK population^27^; includes information on age, sex, and socioeconomic status; and has been validated for multiple diagnoses.^7,28,29,30,31^ THIN is well recognized as a validated epidemiologic resource for medication use.^29^ We previously validated diagnostic codes for abdominal operations and ARCs, including SBOs and lysis of adhesions within THIN.^32^

A second retrospective study was performed using Optum’s Clinformatics Data Mart database (Optum), a medical claims database from a single private payer with patient-level data on more than 77 million unique patients from January 1, 2000, to December 31, 2016, within the US. Optum contains inpatient, outpatient, and surgical claims and pharmacy data and has been used for numerous pharmacoepidemiologic studies.^33,34,35,36^

Data analysis was performed from September 1, 2012, to November 24, 2020. This study was approved and/or considered exempt by the University of Pennsylvania and University of Colorado institutional review boards and was approved by THIN’s Scientific Review Committee. Because this study was a secondary analysis, informed consent was not required. All data were deidentified. The study followed the Strengthening the Reporting of Observational Studies in Epidemiology (STROBE) reporting guideline.

### Study Population

The first cohort study included individuals 18 years or older registered in THIN who were undergoing intra-abdominal surgery after cohort entry. Recording of prevalent or historical events may occur in primary care–derived data sets during a patient’s initial visits. Therefore, index intra-abdominal operations were defined as a diagnostic code for an intra-abdominal operation occurring at least 1 year after cohort entry, without a documented surgical code within the first year or before enrollment. This 1-year window has previously been validated for distinguishing between incident and prevalent events.^37^ Surgical diagnostic codes were identified using a previously defined list of 1435 procedural codes within the Read coding system, which identifies medical diagnoses and procedures in THIN (eTable 1 in the Supplement).^32,38^ These codes included any surgical procedure that involved the peritoneum, colon, small bowel, appendix, stomach, esophagus, diaphragm, kidneys, hepatobiliary system, pancreas, female reproductive tract, and intra-abdominal vasculature. Individuals with a diagnosis of inflammatory bowel disease at any time were excluded because of their increased risk of obstruction-related events unrelated to adhesions. Individuals with a history of SBOs or lysis of adhesions before the index surgery were also excluded.

The second cohort study was conducted using data from Optum for the years 2000 to 2016, using the same inclusion and exclusion criteria in THIN. Index intra-abdominal operations were identified using *International Classification of Diseases, Ninth Revision* (*ICD-9*) and *International Statistical Classification of Diseases and Related Health Problems, Tenth Revision *(*ICD-10)* codes and *Current Procedural Terminology* (*CPT*) (version 4) codes for intraperitoneal surgical events (eTable 2 in the Supplement).

### Study Outcome

The primary outcome was any ARC that occurred after a surgical event. In THIN, these events were identified via codes for SBO, lysis of adhesions, or the presence of adhesions from the Read coding system (eTable 1 in the Supplement). These outcomes have previously been validated within THIN.^32^ Within Optum, ARCs were identified using *ICD-9*, *ICD-10*, and *CPT* codes for these outcomes. Because ARCs can occur up to many years after surgery,^5^ outcomes could occur any time during follow-up.

### Variables of Interest

The primary exposure was statin use, defined as receipt of 2 prescriptions for a statin before the surgery. Statin use was defined based on refill prescriptions and total dispensations and coded as a categorical variable as no use, former use (defined as a prescription for statin use coupled with cessation or no further refills encompassing the surgical date of interest), and current use (≥2 prescriptions with a prescribed supply sufficient to include the surgical date). Indications for initiation or cessation of statin use were not available, although statins are most often prescribed for hyperlipidemia or cardiac protection. Statin exposure at the time of surgery was further classified by strength of low-density lipoprotein cholesterol (LDL-C)–lowering effect as low intensity, moderate intensity, and high intensity (see Sensitivity and Secondary Analyses section).^39^

Covariates included age at surgery, sex, and whether the index surgery involved the small bowel or colon. Age was stratified by 3 categories: 18 through 44 years, 45 through 64 years, and 65 years or older. Given the hypothesized role of tissue hypoxia in adhesion formation, hypertension (defined via diagnostic codes or associated medications), hyperlipidemia (via diagnostic codes or measured LDL-C >190 mg/dL [to convert to millimoles per liter, multiply by 0.0259]^40^), obesity (using diagnostic codes or body mass index when available), and tobacco use (using validated diagnostic codes^41^) were also assessed. Last, a diagnosis of malignant tumor that involved the peritoneum before surgery was assessed because subsequent obstruction could be caused by tumor progression rather than adhesions.

### Statistical Analysis

#### Primary Analysis

Statistical analyses were conducted using Stata statistical software, version 16 (StataCorp, LLC). Categorical variables were summarized as ratios and percentages, whereas continuous variables were reported using medians and interquartile ranges (IQRs), stratified by statin use at the time of surgery. Descriptive statistics were used to compare statin users with nonusers and those with ARCs with those without.

The association between statin use relative to no use and ARCs was assessed using time-to-event analyses. Follow-up time started at the time of index intra-abdominal surgery and ended on the earliest of the following: an outcome of interest (ARC) or a censoring event, such as transfer out of a THIN practice or end of coverage period in Optum, death, or the end of recorded data. Because individuals could have multiple periods of coverage with associated gaps in Optum, they were censored after their initial enrollment period. Multivariable Cox proportional hazards regression models were used to assess the association between covariates and ARCs, calculating hazard ratio (HR) point estimates and 95% CIs.^42,43^ Fully adjusted models were inclusive of all variables. Iterative stepwise backward elimination was performed, removing variables not modifying the association between statin use and ARCs by greater than 10% to identify parsimonious models.^44^ Nonproportionality assumptions were assessed using graphical methods and Schoenfeld residuals.^45,46^ All analyses were conducted separately within THIN and Optum.

#### Sensitivity and Secondary Analyses

To assess whether timing of statin use was associated with ARCs and whether individuals prescribed statins were inherently at decreased risk for adhesion formation, a model assessed the associations with ARCs of those who had started taking a statin but stopped using it before their index surgery (ie, former statin use) in THIN and Optum. To assess whether the treatment effect was associated with the HMG-CoA reductase inhibition vs lipid lowering, fibrate use was assessed in both cohorts. To assess the impact of statin potency, statin use was stratified by level of HMG-CoA reductase inhibition in THIN. Statins were grouped based on expected percentage of LDL-C reduction, with low-intensity statins reducing LDL-C by less than 30%, moderate-intensity statins by 30% to 50%, and high-intensity statins by greater than 50%.^39^ Angiotensin-converting enzyme inhibitors (ACEIs) and angiotensin receptor blockers (ARBs) were assessed as an alternative medication class with known antifibrotic effects^47,48,49^ in THIN as well. Because age was a 3-level categorical variable in our primary analyses, we performed our analyses again with the inclusion of age as a continuous variable in THIN. We performed additional sensitivity analyses that examined the association between statins and SBO given the clinical consequences of this specific outcome (eMethods, eResults, and eFigure in the Supplement). To assess whether geographic or temporal trends in surgical approach or statin use influenced the association between statin use and ARCs, we performed our analyses again with the inclusion of year of surgery and country of surgery in THIN. We also assessed specific windows of time for the outcome to have occurred, from 1 to 5 years from the index surgery (eMethods and eResults in the Supplement). E-values were calculated for statin HRs in THIN and Optum to measure the strength of an association of an unmeasured confounder that would be required to bias the HR for statins to the null^50^ (eMethods and eResults in the Supplement). These sensitivity analyses were conducted using the parsimonious multivariable Cox proportional hazards regression model derived during primary analyses. Finally, to ensure we were comparing individuals of similar baseline characteristics at the time of surgery, we performed a propensity score analysis using 100 iterations of 1:1 caliper-based matching (eMethods and eResults in the Supplement).

## Results

### Statin Use, Comorbidities, and ARCs in the THIN Cohort

Within THIN, 148 601 individuals (mean [SD] age at the time of surgery, 49.6 [17.7] years; 70.1% female) who met the inclusion and exclusion criteria underwent an index intra-abdominal operation. Median follow-up time was 1412 days (IQR, 598-2632 days). Baseline characteristics are presented stratified by statin exposure in Table 1 and stratified by outcomes in eTable 3 in the Supplement. The prevalence of comorbidities of interest was consistent with previously published rates within the United Kingdom. A total of 115 291 intra-abdominal operations (77.6%) did not involve the small bowel or colon. A total of 2060 individuals (1.4%) had an ARC during follow-up.

**Table 1.** Characteristics of THIN and Optum Cohorts, Stratified by Statin Exposure^a^

Characteristic	THIN				Optum			
	Non–statin user at the time of surgery		Statin use at the time of surgery		Non–statin user at the time of surgery		Statin use at the time of surgery	
	No. (%)	Incidence rate of ARCs (95% CI)	No. (%)	Incidence rate of ARCs (95% CI)	No. (%)	Incidence rate of ARCs (95% CI)	No. (%)	Incidence rate of ARCs (95% CI)
Sex								
Male	34 671 (78.0)	0.41 (0.38-0.45)	9779 (22.0)	0.49 (0.42-0.57)	271 279 (83.3)	1.32 (1.30-1.34)	54 260 (16.7)	1.56 (1.51-1.62)
Female	96 553 (92.7)	0.24 (0.22-0.25)	7598 (7.3)	0.37 (0.30-0.44)	788 712 (91.4)	1.33 (1.32-1.35)	73 966 (8.6)	1.60 (1.55-1.65)
Age group, y								
18-44	66 506 (98.9)	0.18 (0.17-0.20)	719 (1.1)	0.15 (0.06-0.37)	456 955 (97.9)	1.22 (1.20-1.24)	9799 (2.1)	1.29 (1.17-1.42)
45-64	40 975 (87.5)	0.28 (0.25-0.30)	5878 (12.6)	0.32 (0.25-0.40)	461 571 (87.2)	1.19 (1.17-1.20)	68 033 (12.8)	1.34 (1.29-1.39)
≥65	23 743 (68.8)	0.63 (0.58-0.68)	10 780 (31.2)	0.53 (0.46-0.61)	141 465 (73.7)	2.12 (2.08-2.16)	50 394 (26.3)	2.00 (1.93-2.07)
Hypertension	14 775 (63.6)	0.41 (0.37-0.46)	8466 (36.4)	0.38 (0.32-0.46)	248 854 (76.5)	1.78 (1.75-1.81)	76 658 (23.5)	1.79 (1.74-1.84)
Diabetes	6242 (52.3)	0.35 (0.29-0.44)	5693 (47.7)	0.35 (0.28-0.44)	125 424 (72.2)	1.95 (1.91-2.00)	48 276 (27.8)	1.88 (1.81-1.95)
Obesity	10 264 (79.7)	0.25 (0.21-0.31)	2614 (20.3)	0.36 (0.25-0.51)	141 455 (83.9)	1.87 (1.83-1.92)	27 214 (16.1)	1.89 (1.80-1.98)
Hyperlipidemia	329 (32.7)	0.88 (0.47-1.63)	678 (67.3)	0.29 (0.14-0.61)	375 209 (75.6)	1.50 (1.48-1.53)	120 790 (24.4)	1.60 (1.56-1.63)
Tobacco use	76 209 (85.4)	0.31 (0.29-0.33)	13 024 (14.6)	0.47 (0.41-0.53)	90 978 (85.2)	1.92 (1.87-1.97)	15 816 (14.8)	2.07 (1.95-2.21)
Cancer history	12 886 (76.5)	0.88 (0.80-0.97)	3964 (23.5)	0.80 (0.68-0.98)	103 811 (81.9)	2.68 (2.63-2.74)	22 924 (18.1)	2.71 (2.59-2.83)
Surgery								
Bowel	28 526 (85.6)	0.57 (0.53-0.61)	4784 (14.4)	0.91 (0.78-1.10)	147 720 (87.1)	2.32 (2.28-2.37)	21 801 (12.9)	3.02 (2.89-3.16)
Nonbowel	102 698 (89.1)	0.21 (0.20-0.22)	12 593 (10.9)	0.27 (0.22-0.32)	912 271 (89.6)	1.18 (1.17-1.19)	106 425 (10.4)	1.31 (1.28-1.35)
Fibrate use	305 (35.1)	0.97 (0.50-1.86)	565 (64.9)	0.24 (0.10-0.58)	4558 (60.6)	1.67 (1.47-1.90)	2967 (39.4)	1.99 (1.70-2.32)
During follow-up								
ARCs	1779 (86.4)	0.27 (0.27-0.29)	281 (13.6)	0.43 (0.38-0.49)	47 414 (87.6)	1.33 (1.32-1.34)	6722 (12.4)	1.59 (1.55-1.62)
SBOs^b^	1779 (83.1)	0.19 (0.18-0.21)	281 (16.9)	0.39 (0.34-0.44)	24 866 (84.7)	0.69 (0.68-0.69)	4498 (15.3)	1.05 (1.02-1.08)

Abbreviations: ARCs, adhesion-related complications; Optum, Optum’s Clinformatics Data Mart; SBO, small-bowel obstruction; THIN, The Health Improvement Network.

^a^ Baseline characteristics for those in THIN and Optum using a statin at the time of surgery compared with those who did not use a statin at the time of surgery. Incidence rates are for any ARC after surgery, presented per 100 person-years.

^b^ SBOs are a subgroup of ARCs.

Overall, 11.7% of individuals were using a statin at the time of surgery (Table 1). Median duration of preoperative statin use was 3.7 years (IQR, 1.7-6.5 years). Altogether, 2.3% of individuals had used a statin but had stopped taking the medication before surgery. Fibrate use was less common (0.6%), whereas ACEIs or ARBs were used by 12.6% of the cohort at the time of surgery (eTable 3 in the Supplement).

In multivariable analyses, statin use at the time of intra-abdominal surgery was associated with a reduced hazard of postoperative ARCs (HR, 0.85; 95% CI, 0.74-0.99). Increasing age (HR for individuals aged >65 years, 2.34; 95% CI, 2.07-2.66), bowel surgery (HR, 2.16; 95% CI, 1.97-2.38), and a history of malignant tumors (HR, 1.81; 95% CI, 1.62-2.03) were associated with increased ARC risk (Table 2). Sex, diabetes, hyperlipidemia, and obesity were not associated with ARCs. In our parsimonious model, statin use remained significantly associated with a reduced hazard of ARCs (HR, 0.81; 95% CI, 0.73-0.96).

**Table 2.** Association Between Statin Use and Covariates With Adhesion-Related Complications Within THIN and Optum

Exposure	HR (95% CI)		
	THIN		Optum (fully adjusted model)^b^
	Completely adjusted model	Parsimonious model^a^	
Female sex	1.01 (0.92-1.11)	NA	0.79 (0.77-0.80)
Age group, y			
18-44	1 [Reference]	1 [Reference]	1 [Reference]
45-64	1.47 (1.31-1.65)	1.45 (1.29-1.62)	0.94 (0.92-0.96)
≥65	2.34 (2.07-2.66)	2.27 (2.01-2.56)	1.37 (1.34-1.41)
Hypertension	0.90 (0.80-1.02)	NA	1.21 (1.19-1.24)
Diabetes	0.85 (0.71-1.01)	NA	1.17 (1.14-1.20)
Hyperlipidemia	1.21 (0.75-1.96)	NA	0.95 (0.93-0.97)
Obesity	0.94 (0.79-1.12)	NA	1.28 (1.25-1.31)
Tobacco use	1.22 (1.11-1.34)	1.21 (1.11-1.33)	1.31 (1.27-1.34)
Surgical site involving the bowel	2.16 (1.97-2.38)	2.17 (1.97-2.38)	1.87 (1.83-1.91)
History of malignant tumor	1.81 (1.62-2.03)	1.81 (1.61-2.02)	1.82 (1.78-1.86)
Statin use at the time of surgery	0.85 (0.74-0.99)	0.81 (0.73-0.96)	0.92 (0.90-0.95)

Abbreviations: HR, hazard ratio; NA, not applicable; Optum, Optum’s Clinformatics Data Mart; THIN, The Health Improvement Network.

^a^ Completely adjusted models are presented without backward stepwise elimination. Parsimonious models were created through iterative stepwise backward elimination, with remaining covariates.

^b^ In Optum, the fully adjusted model was identical to the parsimonious model. Backward elimination was not performed.

### Statin Use, Comorbidities, and ARCs in the Optum Cohort

In Optum, 1 188 217 individuals (mean [SD] age at the time of surgery, 48.2 [16.4] years; 72.6% female) with an index intra-abdominal surgery were identified. Within this cohort, 54 136 individuals (4.6%) experienced a postoperative ARC. The median follow-up time was 1227 days (IQR, 515-1647). In all, 10.8% of individuals were using statins at the time of surgery, whereas 0.4% were using fibrates. Rates of comorbidities associated with microvascular disease, history of malignant tumors, and surgical site are described in Table 1. Baseline characteristics of the Optum cohort, stratified by outcome, are presented in eTable 5 in the Supplement.

In this cohort, statin use at the time of surgery was associated with a decreased risk of ARCs (HR, 0.92; 95% CI, 0.90-0.95) (Table 2). Diseases affecting the microvasculature, including hypertension (HR, 1.21; 95% CI, 1.19-1.24), diabetes (HR, 1.17; 95% CI, 1.14-1.20), and obesity (HR, 1.28; 95% CI, 1.25-1.31), bowel operations (HR, 1.87; 95% CI, 1.83-1.91), a history of malignant tumors (HR, 1.82; 95% CI, 1.78-1.86), tobacco use (HR, 1.31; 95% CI, 1.27-1.34), and advanced age (age >65 years; HR, 1.37; 95% CI, 1.34-1.41) were associated with an increased risk of ARCs (Table 2).

### Secondary and Sensitivity Analyses

Fibrates and ACEIs or ARBs were not associated with the risk of ARCs (Table 3 and eTable 7 in the Supplement). When examining those who used statins before surgery but had stopped use of the medication, former statin use was not associated with the risk of ARCs (HR, 1.14; 95% CI, 0.86-1.53), whereas concurrent use remained associated (HR, 0.84; 95% CI, 0.72-0.97) in THIN (Table 3). Similar findings were appreciated in Optum (eTable 7 in the Supplement). Statins were associated with a reduced hazard of SBOs (THIN: HR, 0.80; 95% CI, 0.70-0.92, Optum: HR, 0.88; 95% CI, 0.85-0.91) (eMethods and eTables 4 and 6 in the Supplement).

**Table 3.** Multivariable Model Assessing the Association Between Former Statin Use, Fibrate Use, and ACEI or ARB Use and ARCs and SBO After Surgery in The Health Improvement Network

Exposure	Parsimonious model, HR (95% CI)^a^	
	Any ARC	SBO
Statin user		
Never	1 [Reference]	1 [Reference]
Former	1.14 (0.86-1.53)	1.15 (0.85-1.56)
At the time of surgery		
Statin use	0.84 (0.72-0.97)	0.83 (0.71-0.96)
ACEI or ARB use	0.92 (0.80-1.05)	0.93 (0.81-1.08)
Fibrate use	1.04 (0.61-1.78)	1.11 (0.63-1.93)
Age group, y		
18-44	1 [Reference]	1 [Reference]
45-64	1.45 (1.30-1.63)	3.72 (3.16-4.39)
≥65	2.28 (2.02-2.58)	6.30 (5.32-7.47)
Tobacco use	1.21 (1.10-1.33)	1.21 (1.09-1.35)
Surgical site involving the bowel	2.16 (1.97-2.37)	2.86 (2.57-3.18)
History of malignant tumor	1.81 (1.61-2.02)	1.74 (1.54-1.96)

Abbreviations: ACEI, angiotensin-converting enzyme inhibitor; ARB, angiotensin receptor blocker; ARC, adhesion-related complication; HR, hazard ratio; SBO, small-bowel obstruction.

^a^ Parsimonious model developed via backward elimination in primary analyses. Additional covariates were added to this multivariable model.

Compared with no statin use, use of statins with an expected LDL-C lowering of less than 30% was not associated with a reduction in ARCs (HR, 0.95; 95% CI, 0.68-1.32), whereas those with 30% to 50% LDL-C lowering (HR, 0.78; 95% CI, 0.67-0.91) and greater than 50% LDL-C lowering (HR, 0.77; 95% CI, 0.52-1.14) were significantly associated with reduced ARCs. Similar results were seen for SBOs (eTable 8 in the Supplement).

No association was found between ACEI or ARB use and ARCs (eTable 7 in the Supplement). When age as a continuous covariate was adjusted for, statins remained significantly associated with a reduction in ARCs (HR, 0.81; 95% CI, 0.71-0.93). Adjustment for year and country in THIN did not alter the association between statins and ARCs or SBOs (eTable 9 in the Supplement). The association between statins and ARCs and SBOs remained similar when considering events from 1 to 5 years after surgery (eTable 10 in the Supplement). When propensity score analyses with 1:1 matching was performed, statins remained associated with a decreased hazard of ARCs (HR, 0.41; 95% CI, 0.23-0.74) (eMethods in the Supplement).

## Discussion

Approximately 5% to 15% of patients undergoing intra-abdominal surgery experience a postoperative ARC within 5 years.^5^ Adhesion-related complications result in significant financial and medical comorbidity.^51^ Although the past 2 decades have led to an improved understanding of the pathophysiology of adhesion formation,^52^ preventive options are currently limited. The association of laparoscopy or barrier agent use with ARCs appears limited, and standardized rates of these complications have not decreased.^18^ Identifying avenues for directly targeting profibrotic cytokines may hold promise for preventing ARCs.

Prior animal and in vitro models^23,24,53,54^ have shown that statins downregulate key fibrosis-related cytokines and reduce adhesion formation. These findings have not been evaluated in humans.^25,26^ In the THIN and Optum cohort studies, these previous laboratory findings are translated into the human population, supporting a role for statins in directly inhibiting adhesion formation. With the use of 2 large, population-representative data sets in the UK and US, this analysis found that statin use at the time of intra-abdominal surgery is associated with reduced rates of ARCs, after adjusting for covariates that may influence the risk of these complications. This finding persisted in multiple sensitivity analyses, including examining the most clinically important outcome of SBO and demonstrating that only concurrent statin use was associated with a reduction in ARCs. Although the primary analysis examined this association across all eligible adults to maximize generalizability, the propensity score analysis demonstrated even stronger potential effect estimates.

This research has significant clinical implications. An association between a novel, well-tolerated, and US Food and Drug Administration–approved therapy and a reduced risk of ARCs in those undergoing intra-abdominal surgery was identified. These findings set the stage for future prospective clinical trials assessing the association of statins with ARCs in those undergoing elective surgery. Because statins are inexpensive with excellent safety profiles, their use would likely be cost-effective even with modest efficacy.

### Strengths and Limitations

This research has several strengths. The use of 2 separate populations demonstrates reproducibility of the findings in 2 different health care systems from 2 different countries. These data sets are well validated for pharmacologic exposures. Our research team has validated surgical interventions and ARCs in THIN. The multiple sensitivity analyses confirmed the association appreciated in the primary model. In addition, a dose response for statin use was found, confirming the findings in moderate- to high-intensity statin dosing but not for their lower-intensity counterparts.

As with any pharmacoepidemiologic study, there are several caveats to consider. Misclassification bias is a concern with retrospective data, particularly with claims-based data sets, which are primarily used for billing. However, THIN data are directly derived from the electronic medical records and have been validated for numerous medical conditions,^7,28,29,30,31^ operations, and ARCs.^32^

Residual confounding is a potential concern. Although factors associated with adhesion formation and comorbid medical conditions were adjusted for, findings were confirmed in 2 cohorts, and several sensitivity analyses were performed (including former vs current statin use), 2 negative controls were used, a likely dose-response curve was assessed, and propensity score matching was performed, it is possible that unmeasurable factors may influence the association between statins and ARCs. Such factors could include perioperative complications or medication use, barrier use, and operative techniques. Future prospective research with granular data collection on surgical factors that may augment the association between statins and ARCs is required. The research presented here supports future prospective efforts.

## Conclusions

In summary, this research suggests that statin use may be significantly associated with a decreased probability of ARCs and SBOs after intra-abdominal surgery. Future prospective randomized clinical trials of this inexpensive, well-tolerated therapy will be required to confirm these findings.

## Notes

Supplement. **eMethods and eResults.** Supplemental Methods and Results **eTable 1.** Diagnostic Codes Used to Identify Surgical Events Within the Abdomen in The Health Improvement Network **eTable 2.** Diagnostic Codes Used to Identify Surgical Events in Optum Clinformatics **eTable 3.** Baseline Characteristics of the Study Cohort Within THIN Stratified by the Presence of Adhesion-Related Complications **eTable 4.** Association Between Statin Use and Covariates With Small Bowel Obstruction in THIN **eTable 5.** Baseline Characteristics of Surgical Cohort Within Optum, Stratified by the Presence of Adhesion-Related Complications **eTable 6.** Association Between Statin Use and Covariates With Small Bowel Obstruction in Optum **eTable 7.** Multivariable Model Assessing the Association Between Former Statin Use, Fibrate Use, and Adhesion-Related Complications (ARCs) and Small Bowel Obstruction (SBO) After Surgery in Optum's Clinformatics® Data Mart **eTable 8.** Multivariable Model Assessing the Association Between Statin Dose Intensity and Adhesion-Related Complications (ARCs) and Small Bowel Obstruction (SBO) After Surgery in The Health Improvement Network **eTable 9.** Multivariable Model Assessing the Association Between Statin Use at the Time of Surgery and Adhesion-Related Complications (ARCs) and Small Bowel Obstruction (SBO) After Surgery in The Health Improvement Network, Adjusting for Year of Initial Surgery and Country of Origin **eTable 10.** Association Between Statin Use at the Time of Surgery and ARCs and SBOs Occurring Within 5, 4, 3, 2, and 1 Years of the Incident Surgical Event **eFigure.** Covariate Balance With 1:1 Matching in Propensity Score Analyses **eReferences**
