# Supplementary material for: Association Between Statin Use at the Time of Intra-abdominal Surgery and Postoperative Adhesion-Related Complications and Small-Bowel Obstruction
Source: JAMA Netw Open. 2021 Feb 3;4(2):e2036315. doi: 10.1001/jamanetworkopen.2020.36315 (PMC7859844; doi:10.1001/jamanetworkopen.2020.36315)
Supplement: Supplement. — eMethods and eResults. Supplemental Methods and Results eTable 1. Diagnostic Codes Used to Identify Surgical Events Within the Abdomen in The Health Improvement Network eTable 2. Diagnostic Codes Used to Identify Surgical Events in Optum Clinformatics eTable 3. Baseline Characteristics of the Study Cohort Within THIN Stratified by the Presence of Adhesion-Related Complications eTable 4. Association Between Statin Use and Covariates With Small Bowel Obstruction in THIN eTable 5. Baseline Characteristics of Surgical Cohort Within Optum, Stratified by the Presence of Adhesion-Related Complications eTable 6. Association Between Statin Use and Covariates With Small Bowel Obstruction in Optum eTable 7. Multivariable Model Assessing the Association Between Former Statin Use, Fibrate Use, and Adhesion-Related Complications (ARCs) and Small Bowel Obstruction (SBO) After Surgery in Optum's Clinformatics® Data Mart eTable 8. Multivariable Model Assessing the Association Between Statin Dose Intensity and Adhesion-Related Complications (ARCs) and Small Bowel Obstruction (SBO) After Surgery in The Health Improvement Network eTable 9. Multivariable Model Assessing the Association Between Statin Use at the Time of Surgery and Adhesion-Related Complications (ARCs) and Small Bowel Obstruction (SBO) After Surgery in The Health Improvement Network, Adjusting for Year of Initial Surgery and Country of Origin eTable 10. Association Between Statin Use at the Time of Surgery and ARCs and SBOs Occurring Within 5, 4, 3, 2, and 1 Years of the Incident Surgical Event eFigure. Covariate Balance With 1:1 Matching in Propensity Score Analyses eReferences [file jamanetwopen-e2036315-s001.pdf]

Supplementary Online Content

Scott FI, Vajravelu RK, Mamtani R, et al. Association between statin use at the time of intra-abdominal surgery and postoperative adhesion-related complications and small-bowel obstruction. *JAMA Netw Open*. 2021;4(2):e2036315. doi:10.1001/jamanetworkopen.2020.36315

eMethods and eResults. Supplemental Methods and Results

eTable 1. Diagnostic Codes Used to Identify Surgical Events Within the Abdomen in The Health Improvement Network

eTable 2. Diagnostic Codes Used to Identify Surgical Events in Optum Clinformatics

eTable 3. Baseline Characteristics of the Study Cohort Within THIN Stratified by the Presence of Adhesion-Related Complications

eTable 4. Association Between Statin Use and Covariates With Small Bowel Obstruction in THIN

eTable 5. Baseline Characteristics of Surgical Cohort Within Optum, Stratified by the Presence of Adhesion-Related Complications

eTable 6. Association Between Statin Use and Covariates With Small Bowel Obstruction in Optum

eTable 7. Multivariable Model Assessing the Association Between Former Statin Use, Fibrate Use, and Adhesion-Related Complications (ARCs) and Small Bowel Obstruction (SBO) After Surgery in Optum's Clinformatics® Data Mart

eTable 8. Multivariable Model Assessing the Association Between Statin Dose Intensity and Adhesion-Related Complications (ARCs) and Small Bowel Obstruction (SBO) After Surgery in The Health Improvement Network

eTable 9. Multivariable Model Assessing the Association Between Statin Use at the Time of Surgery and Adhesion-Related Complications (ARCs) and Small Bowel Obstruction (SBO) After Surgery in The Health Improvement Network, Adjusting for Year of Initial Surgery and Country of Origin

eTable 10. Association Between Statin Use at the Time of Surgery and ARCs and SBOs Occurring Within 5, 4, 3, 2, and 1 Years of the Incident Surgical Event

eFigure. Covariate Balance With 1:1 Matching in Propensity Score Analyses

eReferences

This supplementary material has been provided by the authors to give readers additional information about their work.

© 2021 Scott FI et al. *JAMA Network Open.*

eMethods and eResults. Supplemental Methods and Results

Small Bowel Obstruction analyses

A major clinical sequela of adhesive-related complications is small bowel obstruction (SBO). Further, approximately 70% of SBO admissions are thought to be secondary to adhesion formation. While SBOs were included in the overall adhesion-related complication used in the primary analyses, a secondary analysis was performed examining this specific outcome alone.
All other components of these analyses were identical to those described for the primary analysis. These analyses were first conducted in The Health Improvement Network (THIN), and then repeated in Optum's Clinformatics ® Data Mart (Optum).

Propensity score analyses

In order to ensure we were comparing individuals of similar baseline characteristics at the time of surgery, we performed a propensity score analysis using the *pscore* and *psmatch2* routines in Stata within the THIN cohort[1,2], as per our previously described methods[3]. Variables included in propensity score specification included sex, age at the time of surgery, previously recorded comorbidities associated with statin use (obesity, diabetes, hyperlipidemia, and hypertension), smoking, surgical location, and history of malignancy. Multiple methods of matching were ascertained to assess which specification resulted in greatest balance of these factors, including 1:1 and 1:5 caliper matching and kernel weighting. Balance was assessed graphically and statistically using *pstest*.

After exploring these three different matching/weight methods, 1:1 matching was employed given its degree of balance (see Supplemental Figure 1). Within the matched cohort, there were 17,997 individuals. Adjusting for the propensity score in this cohort, statin use at the time of surgery was more strongly associated with a reduction in adhesion-related complications than in our base analyses (adjusted Hazard Ratio 0.41, 95% CI 0.23, 0.74). To ensure this effect estimate was not an artifact induced through matching, we performed 100 iterations of matching, re-sorting the patient population using a randomly generated new patient identifier with each iteration. The average of these 100 hazard ratios was 0.42, the 2.5th percentile 0.24, and the 97.5th percentile 0.62. Only two of 100 iterations were non-significant based on 95% confidence intervals.

Assessing outcomes within specific time windows after the incident surgery

In order to assess if the association between statin use at the time of intra-abdominal surgery and ARCs or SBOs varied depending on the time interval between the surgery of interest and outcomes, we repeated our analyses using our parsimonious model within THIN and only considering outcomes that occurred within specific time-from-surgery intervals as valid. We assessed outcomes occurring within 5, 4, 3, 2, or 1 year of the index surgical event for each of these analyses. The association between statins and ARCs or SBOs remained similar when only considering outcomes within each of these time windows as true outcomes (See Supplemental Table 10).

E-value estimation for the hazard ratio of ARCs with statin use.

E-values have been a suggested means for estimating the potential effect estimate an unmeasured confounder would need to have with both the exposure of interest (statins) and outcome (ARCs) to bias the measured effect estimate to 1.0[4]. These were calculated using Stata's *evalue* package, using the methods described by VanderWeele and Ding. In THIN, the e-value was 1.77, and in Optum it was 1.40

© 2021 Scott FI et al. *JAMA Network Open.*

eTable 1. Diagnostic codes used to identify surgical events within the abdomen in The Health Improvement Network

READ code	Description of code
76z..00	Upper digestive tract operations NOS
76y..00	Other specified operations on upper digestive tract
7710.11	Panproctocolectomy
7714300	Transverse colectomy and ileostomy HFQ
771E000	Open colonoscopy
7717411	Mikulicz colectomy and colostomy
7715y00	Other specified excision of left hemicolon
771F411	Moschowitz sigmoidopexy
7710100	Panproctocolectomy anast ileum to anus & pouch creation HFQ
7710300	Proctocolectomy NEC
771D200	Open reduction of volvulus of sigmoid colon
771Bz11	Colostomy NEC
7716100	Sigmoid colectomy and anastomosis of colon to rectum
771Ay11	Weir appendicostomy
771A000	Tube caecostomy
7715100	Left hemicolectomy+end to end anastomosis of colon to colon
771T000	Sub ex colon rectum creation colon pouch anastom colon anus
7710y00	Other specified total excision of colon and rectum
7719300	Bypass of colon by anastomosis of transverse colon to rectum
771E.00	Open endoscopic operations on colon
7712.11	Extended right hemicolectomy
7711200	Total colectomy and ileostomy NEC
7716.11	Sigmoid colectomy
7714.00	Excision of transverse colon
771C.00	Incision of colon
771F000	Open biopsy of lesion of colon
771B.00	Other exteriorisation of colon
7713z00	Other excision of right hemicolon NOS
771z.00	Colon operations or rectal sigmoidoscopy NOS
7711100	Total colectomy, ileostomy & creation of rectal fistula HFQ
7716z00	Excision of sigmoid colon NOS
7714100	Transverse colectomy and anastomosis of ileum to colon
7719000	Bypass of colon by anastomosis of ileum to colon
7717200	Colectomy and anastomosis NEC
7716200	Sigmoid colectomy and anastomosis NEC
7714.11	Transverse colectomy
7719z00	Bypass of colon NOS
7719.00	Bypass of colon
7716400	Sigmoid colectomy and exteriorisation of bowel NEC
7713.00	Other excision of right hemicolon
7714z00	Excision of transverse colon NOS
7718100	Excision of lesion of colon NEC
7717y00	Other specified other excision of colon
7715.11	Left hemicolectomy
771B200	Refashioning of colostomy

© 2021 Scott FI et al. *JAMA Network Open.*

7716000	Sigmoid colectomy+end to end anastomosis of ileum to rectum
771D.00	Intraabdominal manipulation of colon
7719400	Bypass of colon by anastomosis of colon to rectum NEC
7712y00	Other specified extended excision of right hemicolon
7710z00	Total excision of colon and rectum NOS
7711.11	Total colectomy
771Tz00	Subtotal excision of colon NOS
7711z00	Total excision of colon NOS
7712.00	Extended excision of right hemicolon
7713200	Right hemicolectomy and anastomosis NEC
7718.00	Extirpation of lesion of colon
771Ay00	Other specified exteriorisation of caecum
7713000	Right hemicolectomy+end to end anastomosis of ileum to colon
7710.12	Total proctocolectomy
7711011	Hampton ileo-rectal anastomosis
771A500	Appendicocaecostomy
771B100	End colostomy
7717300	Colectomy and ileostomy NEC
7715200	Left hemicolectomy and anastomosis NEC
7717z11	Colectomy NEC
7716300	Sigmoid colectomy and ileostomy however further qualified
7719y00	Other specified bypass of colon
7713.11	Other right hemicolectomy
7717z12	Hemicolectomy NEC
771A011	Allen-Welch caecostomy
771A100	Refashioning of caecostomy
771B.11	Colostomy
7711000	Total colectomy and anastomosis of ileum to rectum
771C011	Drainage of pericolonic tissue
7712000	Extended right hemicolectomy and end to end anastomosis
771D000	Open reduction of intussusception of colon
771D300	Open reduction of volvulus of colon NEC
771B600	Percutaneous endoscopic sigmoid colostomy
771Ey00	Other specified open endoscopic operation on colon
7712100	Extended right hemicolectomy and anastomosis ileum to colon
771D400	Open relief of strangulation of colon
7713300	Right hemicolectomy and ileostomy however further qualified
771B300	Closure of colostomy
771Fz00	Other open operation on colon NOS
771F300	Open removal of foreign body from colon
7713011	Ileocaecal resection
7710200	Panproctocolectomy and anastomosis of ileum to anus NEC
7717000	Colectomy and end to end anastomosis of colon to colon NEC
771B500	Reduction of prolapse of colostomy
7715.00	Excision of left hemicolon
771..00	Colon operations and sigmoidoscopy of rectum
7711.00	Total excision of colon
7712200	Extended right hemicolectomy and anastomosis NEC

© 2021 Scott FI et al. *JAMA Network Open.*

771A400	Creation of antegrade continence enema stoma
771Ty00	Other specified subtotal excision of colon
7712z00	Extended excision of right hemicolon NOS
771Dy00	Other specified intraabdominal manipulation of colon
7713100	Right hemicolectomy+side to side anast ileum to transv colon
7714400	Transverse colectomy and exteriorisation of bowel NEC
7713y00	Other specified other excision of right hemicolon
771By00	Other specified other exteriorisation of colon
7717412	Paul colectomy and colostomy
771T300	Subtotal excision of colon and creation of colonic pouch NEC
771A311	Closure of caecostomy
771C200	Colotomy
771A.00	Exteriorisation of caecum
7715z00	Excision of left hemicolon NOS
7710111	Parks panproctocolectomy, ileoanal anastom & creation pouch
7719100	Bypass of colon by anastomosis of caecum to sigmoid colon
7715400	Left hemicolectomy and exteriorisation of bowel NEC
771F400	Sigmoidopexy
771D100	Open reduction of volvulus of caecum
7717400	Colectomy and exteriorisation of bowel NEC
771Fy00	Other specified other open operation on colon
7714000	Transverse colectomy and end to end anastomosis
7710.00	Total excision of colon and rectum
771..11	Colon and caecum operations
7711y00	Other specified total excision of colon
771F.00	Other open operations on colon
771T.00	Subtotal excision of colon
7710000	Panproctocolectomy and ileostomy
771B400	Dilation of colostomy
7715300	Left hemicolectomy and ileostomy however further qualified
7717.11	Other colectomy
771Bz00	Other exteriorisation of colon NOS
771B000	Loop colostomy
771A200	Closure of caecostomy
7719200	Bypass colon by anastomosis of transverse to sigmoid colon
771Cy00	Other specified incision of colon
771F200	Enterorrhaphy of colon
7716y00	Other specified excision of sigmoid colon
7716.00	Excision of sigmoid colon
7717100	Colectomy and side to side anastomosis of ileum to colon NEC
7718z00	Extirpation of lesion of colon NOS
7717500	Partial colectomy NEC
7715000	Left hemicolectomy+end to end anastomosis of colon to rectum
7717z00	Other excision of colon NOS
771C100	Caecotomy
7714y00	Other specified excision of transverse colon
771C000	Drainage of colon
7712300	Extended right hemicolectomy and ileostomy HFQ

© 2021 Scott FI et al. *JAMA Network Open.*

7718000	Excision of diverticulum of colon
7714200	Transverse colectomy and anastomosis NEC
771Dz00	Intraabdominal manipulation of colon NOS
771B311	Mikulicz closure of colostomy
7717.00	Other excision of colon
7718200	Destruction of lesion of colon NEC
771A.11	Caecostomy
771y.00	Other specified operations on colon or rectal sigmoidoscopy
771E.11	Open colonoscopy
771A300	Caecostomy NEC
7701200	Incidental appendicectomy
7702z00	Other operation on appendix NOS
7702y00	Other specified other operation on appendix
7700400	Endoscopic emergency appendicectomy
7701.11	Non emergency appendicectomy
7701000	Interval appendicectomy
7702000	Drainage of abscess of appendix
7702.00	Other operations on appendix
7700300	Emergency appendicectomy NEC
7700.11	Emergency appendicectomy
7700000	Emergency excision of abnormal appendix and drainage HFQ
770y.00	Other specified operations on appendix
7702100	Drainage of appendix NEC
7700z00	Emergency excision of appendix NOS
7701z00	Other excision of appendix NOS
7701.00	Other excision of appendix
7701400	Endoscopic appendicectomy NEC
7702200	Appendicostomy
7701100	Prophylactic appendicectomy NEC
770..00	Appendix operations
770z.00	Appendix operations NOS
7700200	Emergency excision of normal appendix
7701y00	Other specified other excision of appendix
7700100	Emergency excision of abnormal appendix NEC
7700y00	Other specified emergency excision of appendix
7701300	Planned delayed appendicectomy NEC
7700.00	Emergency excision of appendix
7701z11	Appendicectomy NEC
7722100	Open cauterisation of lesion of rectum
7723.13	Rectopexy
773C500	Closure of bowel fistula
7721015	Abdominoperineal resection of rectum and end colostomy
7720z00	Partial excision of rectum & sigmoid colon for prolapse NOS
7721y00	Other specified excision of rectum
7721500	Ant resect rectum stapled anast sigmoid to anus with J pouch
7721100	Proctectomy and anastomosis of colon to anus
7721.00	Excision of rectum NEC
772..00	Rectum operations

© 2021 Scott FI et al. *JAMA Network Open.*

7720.11	Rectosigmoidectomy
773Ey00	Other specified therapeutic operations on ileoanal pouch
7721z00	Excision of rectum NOS
773C600	Closure of perforated bowel ulcer NEC
773C.00	Other operations on bowel
7720100	Rectosigmoidectomy, closure rectal stump & exteriorise bowel
773E.00	Therapeutic operations on ileoanal pouch
773Cz00	Other operation on bowel NOS
7721013	Miles abdominoperineal excision of rectum and end colostomy
7722000	Open excision of lesion of rectum
7721011	Gabriel abdominoperineal excision of rectum & end colostomy
7723.11	Proctopexy for prolapse of rectum
7722400	Open destruction of lesion of rectum NEC
7721012	Lloyd-Davies abdominoperineal excision rectum+end colostomy
7721000	Abdominoperineal excision of rectum and end colostomy
7721.11	Proctectomy
773E100	Revision of ileoanal pouch
773Ez00	Therapeutic operations on ileoanal pouch NOS
7720000	Rectosigmoidectomy and rectopexy
7721300	Anterior resection of rectum and anastomosis NEC
7720111	Hartmann rectosigmoidectomy and colostomy
7722.00	Open extirpation of lesion of rectum
7722z00	Open extirpation of lesion of rectum NOS
773Cy00	Other specified other operation on bowel
7721200	Anterior resection rectum + staple anastomosis colon-rectum
773E000	Excision of ileoanal pouch
7723.12	Rectopexy for prolapse
7720011	Rectopexy
7722300	Open laser destruction of lesion of rectum
7720y00	Partial excision of rectum and sigmoid colon for prolapse OS
7721400	Anterior resection of rectum and exteriorisation of bowel
7722.11	Open operation on rectal polyp
7723.00	Fixation of rectum for prolapse
7722.12	Open polypectomy of rectum
7721014	Rankin abdominoperineal excision of rectum and end colostomy
7720.00	Partial excision of rectum and sigmoid colon for prolapse
7721600	Perineal resection of rectum HFQ
760L011	Belsey fundoplicatation using thoracic approach
7600111	Roux-en-y oesophagogastrectomy
760L200	Antireflux fundoplication using abdominal approach
7601400	Total oesophagectomy and interposition of colon NEC
7600300	Extended total gastrectomy
760A011	Insertion of Celestin tube into oesophagus via stomach
760K.12	Repair of hiatus hernia
760Ly00	Other specified antireflux operation
7608000	Cardiomyotomy
7600012	Ivor - Lewis oesophagogastrectomy
7600z00	Excision of oesophagus and stomach NOS

© 2021 Scott FI et al. *JAMA Network Open.*

760L100	Antireflux operation using thoracic approach NEC
7605200	Removal of bypass of oesophagus
7606300	Repair of rupture of oesophagus
760L111	Antireflux procedure using thoracic approach NEC
7605y00	Other specified attention to connection of oesophagus
760K000	Repair of oesophageal hiatus using thoracic approach
760K200	Repair of oesophageal hiatus using abdominal approach
760L012	Nissen fundoplication using thoracic approach
7609z00	Open operation on oesophageal varices NOS
7606y00	Other specified repair of oesophagus
7609y11	Tanner devascularisation for bleeding varices
7609100	Transection of oesophagus using staple gun
760Ky00	Other specified repair of diaphragmatic hernia
7603311	Dahlman excision of diverticulum of oesphagus
760L312	Hill repair of hiatus hernia and gastropexy
760A.11	Insertion of Atkinson tube in oesophagus
7606z00	Repair of oesophagus NOS
7602.00	Partial excision of oesophagus
7600013	Oesophgastrectomy
7603300	Excision of diverticulum of oesophagus
7600100	Oesophagogastrect & anastom oesophagus to transposed jejunum
760L800	Laparoscopic Nissen fundoplication using abdominal approach
7601213	Roux oesophagectomy and interposition of jejunal loop
760L000	Antireflux fundoplication using thoracic approach
760L.11	Oesophageal reflux operations
7600.11	Oesophagogastrectomy
760Kz00	Repair of diaphragmatic hernia NOS
7604z00	Bypass of oesophagus NOS
760M000	Revision of fundoplication of stomach
7603000	Excision of lesion of oesophagus
7604500	Bypass of oesophagus by interposition of colon NEC
760K012	Mason repair of oesophageal hiatus hernia
7604300	Bypass of oesophagus by interposition of jejunum NEC
760L311	Antireflux gastroplasty
760A000	Insertion of tubal prosthesis into oesophagus via stomach
7609400	Open injection sclerotherapy to oesophageal varices
760K100	Repair of diaphragmatic hernia using thoracic approach NEC
760B100	Open removal of foreign body from oesophagus
7601z00	Total excision of oesophagus NOS
760..00	Oesophagus (including hiatus hernia) operations
7601.11	Total oesophagectomy
760K.00	Repair of diaphragmatic hernia
7603100	Open laser destruction of lesion of oesophagus
7600011	Bancroft partial gastrectomy and oesophagogastrostomy
7608.00	Incision of oesophagus
7602z00	Partial excision of oesophagus NOS
760A300	Open removal of tubal prosthesis from oesophagus
7606.00	Repair of oesophagus

© 2021 Scott FI et al. *JAMA Network Open.*

7600y00	Other specified excision of oesophagus and stomach
7601111	McKewown total oesophagectomy
760B000	Open biopsy of lesion of oesophagus
7601000	Total oesophagectomy and anastomosis of pharynx to stomach
760A.00	Open placement of prosthesis in oesophagus
7605000	Revision of interposition anastomosis of oesophagus
760L500	Insertion of Angelchick prosthesis
760L.00	Antireflux operations
7604100	Bypass of oesophagus by anastomosis of oesophagus to stomach
7602.11	Partial oesophagectomy
760z.00	Oesophagus (including hiatus hernia) operations NOS
7600.00	Excision of oesophagus and stomach
7602z11	Oesophagectomy NEC
7608100	Oesophagomyotomy NEC
760Lz00	Antireflux operation NOS
760A100	Open insertion of tubal prosthesis into oesophagus NEC
7601y00	Other specified total excision of oesophagus
7608011	Heller cardiomyotomy
760M200	Removal of Angelchick prosthesis
7601.00	Total excision of oesophagus
760K500	Laparoscopic repair of hiatus hernia
7601200	Total oesophagectomy and interposition of jejunum NEC
760..11	Oesophagus operations
7602400	Partial oesophagectomy+interposition microvasc attach colon
760K.11	Repair of oesophageal hiatus hernia
7600000	Oesophagogastrectomy & anastomosis of oesophagus to stomach
7605100	Revision of anastomosis of oesophagus NEC
760L700	Endoscopic Nissen fundoplication using thoracic approach
760L300	Antireflux gastropexy
7608y00	Other specified incision of oesophagus
7602000	Partial oesophagectomy and end to end anastomosis of oesoph
7602y00	Other specified partial excision of oesophagus
7602300	Partial oesophagectomy+anastomosis oesophagus to jejunum NEC
760L611	Thal - Nissen stricturoplasty
760B.00	Other open operations on oesophagus
760y.00	Oesophagus (including hiatus hernia) operations OS
760A200	Open revision of tubal prosthesis in oesophagus
7609000	Disconnection of azygous vein
760By00	Other specified other open operation on oesophagus
760M.00	Revision of antireflux operations
7600200	Oesophagogastrect & anastomosis of oesophagus to jejunum NEC
760Az00	Open placement of prosthesis in oesophagus NOS
7604.00	Bypass of oesophagus
760K300	Repair of diaphragmatic hernia using abdominal approach NEC
760Bz00	Other open operation on oesophagus NOS
7604000	Bypass of oesophagus by anastomosis of oesophag to oesophag
760L211	Nissen fundoplication using abdominal approach
760L600	Oesophagogastric fundoplasty

© 2021 Scott FI et al. *JAMA Network Open.*

7608z00	Incision of oesophagus NOS
7609200	Transection of oesophagus NEC
7609.00	Open operations on oesophageal varices
760Mz00	Revision of antireflux operation NOS
761B000	Pyloromyotomy
761Hz00	Other operation on stomach NOS
7618000	Gastropexy NEC
761C100	Repair of perforation of pylorus
7617z00	Gastrostomy operation NOS
7611211	Billroth II partial gastrectomy and gastroenterostomy
761J000	Closure of perforated gastric ulcer
7619z00	Incision of stomach NOS
761Jy00	Other specified operation on gastric ulcer
7617y00	Other specified gastrostomy operation
7616014	Mayo-Ward gastrojejunostomy
7611500	Sleeve gastrectomy NEC
7614.00	Connection of stomach to duodenum NEC
7616600	Laparoscopic gastric bypass
761J.00	Operations on gastric ulcer
7614z00	Connection of stomach to duodenum NOS
7611700	Wedge resection of stomach
7618.00	Other repair of stomach
7611212	Hofmeister valved gastrectomy
761Cz00	Other operation on pylorus NOS
7613500	Partitioning of stomach NEC
7610100	Total gastrectomy and anastomosis of oesophagus to duodenum
761B300	Revision of pyloroplasty
7616100	Revision of anastomosis of stomach to jejunum NEC
7612.00	Open extirpation of lesion of stomach
761B.00	Incision of pylorus
7610000	Total gastrectomy and excision of surrounding tissue
7610.11	Schlatter total gastrectomy
761J111	Suture of ulcer of stomach NEC
7611000	Partial gastrectomy and anastomosis of stomach to duodenum
761B.11	Pyloromyotomy
7615000	Bypass stomach by anastomosis stomach to transposed jejunum
7617200	Reconstruction of gastrostomy
7611100	Partial gastrectomy & anastomosis stomach to transp jejunum
761A000	Open biopsy of lesion of stomach
7616300	Open reduction of intussusception of gastroenterostomy
7611012	Billroth I partial gastrectomy & gastroduodenal anastomosis
761C.00	Other operations on pylorus
7611.00	Partial excision of stomach
7618y00	Other specified other repair of stomach
7615.11	Gastrojejunostomy, transposed
7613.00	Plastic operations on stomach
7614111	Jaboulay gastroduodenostomy
761B600	Open dilation of pylorus

© 2021 Scott FI et al. *JAMA Network Open.*

7611215	Polya partial gastrectomy and anastomosis stomach to jejunum
7617300	Closure of gastrostomy
761Az00	Other open operation on stomach NOS
7611800	Completion gastrectomy
761A100	Open insertion of prosthesis into stomach
7617.00	Gastrostomy operations
7611A00	Distal partial gastrectomy
7613400	Partitioning of stomach using staples
761A300	Reduction of volvulus of stomach
7617.11	Artificial opening to stomach
7610y00	Other specified total excision of stomach
7618200	Closure of abnormal opening of stomach NEC
7611300	Partial gastrectomy preserving pylorus
761B211	Finney pyloroplasty
7612100	Open excision of lesion of stomach NEC
7618z00	Other repair of stomach NOS
761y.00	Other specified operations on stomach or pylorus
761J100	Closure of gastric ulcer NEC
761By00	Other specified incision of pylorus
7617000	Creation of permanent gastrostomy
7612y00	Other specified open extirpation of lesion of stomach
7614000	Bypass of stomach by anastomosis of oesophagus to duodenum
761..11	Gastric and pylorus operations
761B100	Repair of congenital atresia of pylorus
7610400	Total gastrectomy and anastomosis oesophagus to jejunum NEC
7617112	Creation of Witzel temporary gastrostomy
7611200	Partial gastrectomy and anastomosis stomach to jejunum NEC
7610.00	Total excision of stomach
7611900	Proximal gastrectomy
7616011	Gastroenterostomy NEC
761B200	Pyloroplasty NEC
7617.12	Creation of gastrostomy
7614200	Revision of anastomosis of stomach to duodenum
7610.12	Total gastrectomy
761Bz00	Incision of pylorus NOS
7614100	Bypass of stomach by anastomosis of stomach to duodenum
761..00	Stomach and pylorus operations
7612500	Resection of gastric ulcer by cautery
761A.00	Other open operations on stomach
7612000	Open excision of polyp of stomach
7616.00	Other connection of stomach to jejunum
761z.00	Stomach and pylorus operations NOS
7614y00	Other specified connection of stomach to duodenum
7613200	Laparoscopic adjustable gastric banding
7613100	Partitioning of stomach
7616x00	Conversion from previous anastomosis stomach to jejunum NEC
7616013	Mason high gastric bypass
7613300	Partitioning of stomach using band

7615.00	Connection of stomach to transposed jejunum
761Hy00	Other specified other operation on stomach
7611214	Moynihan partial gastrectomy & retrocolic gastroenterostomy
7615300	Closure of connection of stomach to transposed jejunum
7613111	Mason vertical banded gastroplasty
7613000	Gastroplasty NEC
7617111	Creation of gastrostomy NEC
761Ay00	Other specified other open operation on stomach
7616200	Conversion to anastomosis of stomach to jejunum NEC
761A200	Open insertion of feeding tube into stomach
761C000	Open biopsy of lesion of pylorus
7619.00	Incision of stomach NEC
7619000	Open removal of foreign body from stomach
7615200	Conversion to anastomosis of stomach to transposed jejunum
7616000	Bypass of stomach by anastomosis of stomach to jejunum NEC
7617100	Creation of temporary gastrostomy
7616015	Printer high gastric bypass
761J.11	Stomach ulcer operations
7611z00	Partial excision of stomach NOS
7611y00	Other specified partial excision of stomach
7619100	Gastrotomy and ligation of bleeding point of stomach
7611213	Mayo partial gastrectomy and retrocolic gastroenterostomy
761B500	Pyloromyotomy and wedge resection
7610300	Total gastrectomy & anastomosis oesophagus to transp jejunum
7611.11	Partial gastrectomy
761B011	Ramstedt pyloromyotomy
7611216	Partial gastrectomy and gastrojejunostomy
7616012	Gastrojejunostomy NEC
7611400	Sleeve gastrectomy and duodenal switch
761A400	Devascularisation of stomach
761Cy00	Other specified other operation on pylorus
761H.00	Other operations on stomach
7612200	Open laser destruction of lesion of stomach
761B212	Heinecke-Mikulicz pyloroplasty
7615z00	Connection of stomach to transposed jejunum NOS
7615y00	Other specified connection of stomach to transposed jejunum
761Jz00	Operation on gastric ulcer NOS
761B213	Mikulicz-Heinecke pyloroplasty
7616z00	Other connection of stomach to jejunum NOS
7611600	Laparoscopic sleeve gastrectomy
7610z00	Total excision of stomach NOS
7619.11	Gastrotomy NEC
7618100	Closure of perforation of stomach NEC
7632y00	Other specified jejunostomy
7623500	Creation of duodenostomy
7648000	Open biopsy of lesion of ileum
7633y00	Other specified bypass of jejunum
7623z00	Other open operation on duodenum NOS

© 2021 Scott FI et al. *JAMA Network Open.*

7626z00	Other operation on duodenum NOS
7647100	Open relief of strangulation of ileum
7627.00	Operations on duodenal ulcer
7638z00	Other operation on jejunum NOS
7627000	Closure of perforated duodenal ulcer
7623.00	Other open operations on duodenum
7648300	Closure of perforation of ileum
7623411	Duodenotomy NEC
7621100	Open destruction of lesion of duodenum
7620.11	Duodenectomy
7627100	Suture of duodenal ulcer not elsewhere classified
7635.00	Other open operations on jejunum
7642300	Bypass of ileum by anastomosis of ileum to transverse colon
764z.00	Ileum operations NOS
7627z00	Operation on duodenal ulcer NOS
763z.00	Jejunum operations NOS
7623000	Open biopsy of lesion of duodenum
7620y00	Other specified excision of duodenum
7644100	Closure of anastomosis of ileum
7645212	Creation of split ileostomy
7642100	Bypass of ileum by anastomosis of ileum to ileum
762y.00	Other specified operations on duodenum
7622y00	Other specified bypass of duodenum
7645y00	Other specified creation of ileostomy
7620z11	Duodenectomy NEC
7640300	Ileectomy and anastomosis of ileum to colon
7621z00	Open extirpation of lesion of duodenum NOS
7648z00	Other open operation on ileum NOS
7626y00	Other specified other operation on duodenum
7640z11	Ileectomy NEC
7632200	Closure of jejunostomy
7642400	Bypass of ileum by anastomosis of ileum to colon NEC
7648y00	Other specified other open operation on ileum
7643.11	Other anastomosis of ileum
7622z00	Bypass of duodenum NOS
762..00	Duodenum operations
7635100	Incision of jejunum
7635y00	Other specified other open operation on jejunum
7645z00	Creation of ileostomy NOS
7640.11	Ileectomy
7644z00	Attention to connection of ileum NOS
7621000	Excision of lesion of duodenum
7643z00	Other connection of ileum NOS
7644000	Revision of anastomosis of ileum
7642y00	Other specified bypass of ileum
7644y00	Other specified attention to connection of ileum
7633000	Bypass of jejunum by anastomosis of jejunum to jejunum
7623300	Open insertion of tubal prosthesis into duodenum

© 2021 Scott FI et al. *JAMA Network Open.*

7643000	Anastomosis of ileum to caecum
7642z00	Bypass of ileum NOS
7644200	Resection of ileostomy
7645000	Creation of continent ileostomy
7647311	Noble plication of ileum
7627200	Oversew of blood vessel of duodenal ulcer
7632.11	Artificial opening into jejunum
7630z00	Excision of jejunum NOS
7643400	Anastomosis of ileum to anus and creation of pouch HFQ
7622000	Bypass of duodenum by anastomosis of stomach to jejunum
7638400	Transposition of jejunum
7633212	Jejunocolostomy
7622.00	Bypass of duodenum
7642000	Bypass of ileum by anastomosis of jejunum to ileum
7648700	Enterotomy and removal of gallstone
7630y00	Other specified excision of jejunum
7648400	Exclusion of segment of ileum
764Bz00	Other operation on ileum NOS
7632.00	Jejunostomy
764y.00	Other specified operations on ileum
7640y00	Other specified excision of ileum
764..11	Small intestine operations
7640z00	Excision of ileum NOS
764By00	Other specified other operation on ileum
7643y00	Other specified other connection of ileum
7647.00	Intraabdominal manipulation of ileum
762z.00	Duodenum operations NOS
7632z00	Jejunostomy NOS
7640200	Ileectomy and anastomosis of ileum to ileum
764C.00	Transplantation of ileum
7620.00	Excision of duodenum
7648600	Oversewing of small bowel
7647400	Mobilisation of intestine
7632000	Creation of jejunostomy
7643200	Anastomosis of ileum to colon NEC
7643300	Anastomosis of ileum to rectum
7647300	Plication of ileum
7647z00	Intraabdominal manipulation of ileum NOS
7635200	Closure of perforation of jejunum
7641100	Excision of lesion of ileum NEC
7633z00	Bypass of jejunum NOS
7641000	Excision of Meckel diverticulum
7638100	Intubation of jejunum for measurement of intestinal function
7631000	Excision of lesion of jejunum
7645y11	Creation of Gross ileostomy
7620z00	Excision of duodenum NOS
7642200	Bypass of ileum by anastomosis of ileum to caecum
7648.00	Other open operations on ileum

7645100	Creation of temporary ileostomy
7642.00	Bypass of ileum
7648100	Strictureplasty of ileum
7622200	Bypass of duodenum by anastomosis of duodenum to jejunum
7641z00	Open extirpation of lesion of ileum NOS
7623400	Incision of duodenum NEC
7644.11	Attention to anastomosis of ileum
7640100	Ileectomy and anastomosis of duodenum to ileum
7645.00	Creation of ileostomy
7627y00	Other specified operation on duodenal ulcer
763y.00	Other specified operations on jejunum
7642500	Duodenal switch
7630.11	Jejunectomy
7643.00	Other connection of ileum
7632100	Refashioning of jejunostomy
7640.00	Excision of ileum
7626.00	Other operations on duodenum
7620000	Gastroduodenectomy
7638200	Passage of Crosby capsule into jejunum for biopsy of mucosa
7647000	Open reduction of intussusception of ileum
7643100	Anastomosis of ileum to transverse colon
7644.00	Attention to connection of ileum
7641y00	Other specified open extirpation of lesion of ileum
7635000	Open biopsy of lesion of jejunum
7623100	Closure of perforation of duodenum NEC
7630.00	Excision of jejunum
763..00	Jejunum operations
7633100	Bypass of jejunum by anastomosis of jejunum to ileum
7638y00	Other specified other operation on jejunum
7633200	Bypass of jejunum by anastomosis of jejunum to colon
7634.11	Operative jejunoscopy
7644300	Resection of ileo-colic anastomosis
7645200	Creation of defunctioning ileostomy
7638.00	Other operations on jejunum
7630300	Partial jejunectomy and anastomosis of jejunum to ileum
7641.00	Open extirpation of lesion of ileum
764..00	Ileum operations
7634000	Open jejunoscopy
7622100	Bypass of duodenum by anastomosis of duodenum to duodenum
7633.00	Bypass of jejunum
7803y00	Other specified repair of liver
7803z00	Repair of liver NOS
7802400	Excision of multiple lesions of liver
7806z00	Therapeutic laparoscopic operation on liver NOS
7802y00	Other specified extirpation of lesion of liver
7805200	Exploration of liver
7804100	Open removal of calculus from liver
7804z00	Incision of liver NOS

© 2021 Scott FI et al. *JAMA Network Open.*

7802500	Excision of lesion of liver NEC
7804200	Open wedge biopsy of lesion of liver
7800z00	Transplantation of liver NOS
7803000	Removal of lacerated fragment of liver
7801500	Left hepatic trisegmentectomy
7806y00	Therapeutic laparoscopic operation on liver OS
780..00	Liver operations
7805100	Open insertion of cannula for perfusion of liver
7801y00	Other specified partial excision of liver
7801.00	Partial excision of liver
7800.00	Transplantation of liver
7801100	Left hemihepatectomy
7804.11	Hepatotomy
7807z00	Diagnostic laparoscopic examination of liver NOS
7800200	Replacement of previous liver transplant
7805z00	Other open operation on liver NOS
7806100	Laparoscopic insertion of cannula into gall bladder
7805211	Exploration of liver transplant
7801700	Extended right hemihepatectomy
7801600	Right hepatic trisegmentectomy
7807100	Laparoscopic ultrasound examination liver biop lesion liver
7806.00	Therapeutic endoscopic operations on liver using laparoscope
780y.00	Other specified operations on liver
7803200	Packing of laceration of liver
7802z00	Extirpation of lesion of liver NOS
780z.00	Liver operations NOS
7807200	Laparoscopic ultrasound examination of liver NEC
7801300	Wedge excision of liver
7804y00	Other specified incision of liver
7800100	Heterotopic transplantation of liver
7801400	Marsupialisation of lesion of liver
7801900	Right hemihepatectomy NEC
7800y00	Other specified transplantation of liver
7801.11	Partial hepatectomy
7800000	Orthotopic transplantation of liver
7804.00	Incision of liver
7800111	Auxillary liver transplant
7801z00	Partial excision of liver NOS
7801A00	Left hemihepatectomy NEC
7801200	Resection of segment of liver
7804000	Open drainage of liver
7801000	Right hemihepatectomy
7805.00	Other open operations on liver
7807000	Diagnostic laparoscopic examination and biopsy liver lesion
7803100	Repair of laceration of liver
7805y00	Other specified other open operation on liver
7803.00	Repair of liver
7802000	Excision of lesion of liver

7807.00	Diagnostic endoscopic examination of liver using laparoscope
7807y00	Diagnostic laparoscopic examination of liver OS
7810300	Partial cholecystectomy and exploration of common bile duct
781..00	Gall bladder operations
7812200	Repair of perforation of gall bladder
7811311	Cholecystoenterostomy
7814000	Excision of lesion of gall bladder
7810100	Total cholecystectomy and exploration of common bile duct
7811.00	Connection of gall bladder
7810411	Thorek partial cholecystectomy
7812100	Closure of cholecystotomy
7811100	Anastomosis of gall bladder to duodenum
7811011	Cholecystogastrostomy
7812.00	Repair of gall bladder
7810211	Cholecystectomy NEC
7813200	Drainage of tissue surrounding gall bladder
7810.00	Excision of gall bladder
7811111	Cholecystoduodenostomy
7810z00	Excision of gall bladder NOS
7810500	Endoscopic cholecystectomy
7813000	Open removal of calculus from gall bladder
7811300	Anastomosis of gall bladder to intestine NEC
7812000	Closure of fistula of gall bladder
7810.11	Cholecystectomy
7813012	Open cholelithotomy
7817.00	Other operations on gall bladder
7811211	Cholecystojejunostomy
7812z00	Repair of gall bladder NOS
7813011	Bobb cholelithotomy
7811.11	Anastomosis of gall bladder
7811200	Anastomosis of gall bladder to jejunum
781..11	Cholecyst operations
7813.00	Incision of gall bladder
7811212	Roux-en-y cholecystojejunostomy
7810y00	Other specified excision of gall bladder
7814300	Removal of foreign body from gall bladder
7813111	Cholecystostomy NEC
7810000	Total cholecystectomy and excision of surrounding tissue
7811000	Anastomosis of gall bladder to stomach
7810511	Laparoscopic cholecystectomy
7814200	Exploration of gall bladder
7813y00	Other specified incision of gall bladder
7813.11	Cholecystotomy
7814.00	Other open operations on gall bladder
7810200	Total cholecystectomy NEC
781z.00	Gall bladder operations NOS
7814z00	Other open operation on gall bladder NOS
7813100	Drainage of gall bladder

© 2021 Scott FI et al. *JAMA Network Open.*

7814100	Open biopsy of lesion of gall bladder
7813z00	Incision of gall bladder NOS
7814y00	Other specified other open operation on gall bladder
7811400	Revision of anastomosis of gall bladder
7810400	Partial cholecystectomy NEC
7824400	Roux-en-y procedure for biliary atresia
7825z00	Repair of bile duct NOS
7826000	Open removal of calculus and drainage of bile duct
7826111	Choledocholithotomy NEC
782A300	Operative choledochoscopy NEC
782Ay00	Other specified other open operation on bile duct
7825000	Reconstruction of bile duct
7823300	Revision of anastomosis of common bile duct
7820200	Partial excision and anastomosis of bile duct to jejunum
7822011	Kasai hepatojejunostomy + insertion tubal prosthesis
782y.00	Other specified operations on bile duct
7826.11	Choledochotomy
7826y00	Other specified incision of bile duct
7823200	Anastomosis of common bile duct to jejunum NEC
7823z00	Connection of common bile duct NOS
7826.12	Exploration of bile duct
7823z11	Pancreatectomy NEC
7826.00	Incision of bile duct
7826100	Open removal of calculus from bile duct NEC
7823y00	Other specified connection of common bile duct
7820.00	Excision of bile duct
7823211	Cholodochojejunostomy NEC
7822100	Anastomosis of hepatic duct to jejunum NEC
7823111	Roux-en-y choledochoenterostomy
7824y00	Other specified open operation on prosthesis in bile duct
782z.00	Bile duct operations NOS
7826z00	Incision of bile duct NOS
7825111	Choledochocholedochostomy
7822z00	Connection of hepatic duct NOS
782..00	Bile duct operations
7822y00	Other specified connection of hepatic duct
7821z00	Extirpation of lesion of bile duct NOS
7826300	Exploration of bile duct
7822300	Open dilation of anastomosis of hepatic duct
782A200	Direct puncture operative cholangiography
782A000	Open biopsy of lesion of bile duct
782..11	Choledochus operations
7823000	Anastomosis of common bile duct to duodenum
7825100	Reanastomosis of bile duct
7822200	Revision of anastomosis of hepatic duct
7823400	Open dilation of anastomosis of common bile duct
782Az00	Other open operation on bile duct NOS
7825.11	Choledochoplasty

© 2021 Scott FI et al. *JAMA Network Open.*

7820y00	Other specified excision of bile duct
7823011	Choledochoduodenostomy
7821000	Excision of lesion of bile duct
7823.00	Connection of common bile duct
7825y00	Other specified repair of bile duct
782R.00	Laparoscopic ultrasound examination of bile duct
782Rz00	Laparoscopic ultrasound examination of bile duct NOS
7823.11	Anastomosis of common bile duct
7822.00	Connection of hepatic duct
7820000	Excis ampulla of Vater & replant com bile duct in duodenum
7823100	Anastomosis of common bile duct to transposed jejunum
7824z00	Open operation on prosthesis in bile duct NOS
7825.00	Repair of bile duct
782A.00	Other open operations on bile duct
7822000	Anast hepatic duct to transp jejunum+insert tubal prosth
7821.00	Extirpation of lesion of bile duct
7830100	Transplantation of whole pancreas
7835.00	Connection of pancreatic duct
7835.12	Roux-en-y pancreaticojejunostomy
783D000	Laparoscopic ultrasound examination panc biop lesion panc
7834100	Excision of lesion of pancreas NEC
7835212	Peustow pancreaticojejunostomy
783Fz00	Other operations on pancreas NOS
783D.00	Laparoscopic ultrasound examination of pancreas
7833z00	Other partial excision of pancreas NOS
7833.12	Partial pancreatectomy NEC
7835211	Pancreaticojejunostomy
7833y00	Other specified other partial excision of pancreas
7834.00	Extirpation of lesion of pancreas
7839200	Open pancreatography NEC
7832z00	Excision of head of pancreas NOS
7831z00	Total excision of pancreas NOS
7835200	Anastomosis of pancreatic duct to transposed jejunum
7837000	Open cystogastrotomy of pancreas
783z.00	Pancreas operations NOS
7833500	Pancreatectomy NEC
7831200	Excision of transplanted pancreas
7832.11	Pancreatectomy, head of pancreas
7836400	Open dilation of pancreatic duct
783F.00	Other operations on pancreas
7833000	Subtotal pancreatectomy
7836100	Open removal of calculus from pancreatic duct
7833.00	Other partial excision of pancreas
7832200	Pancreaticoduodenectomy NEC
7831.11	Total pancreatectomy
7832100	Pancreaticoduodenectomy and resection of antrum of stomach
7839300	Exploration of pancreas NEC
783..00	Pancreas operations

© 2021 Scott FI et al. *JAMA Network Open.*

7836.00	Other open operations on pancreatic duct
7835300	Anastomosis of pancreatic duct to jejunum NEC
7831100	Total pancreatectomy NEC
7832113	Whipple pancreaticoduodenectomy
7838.00	Incision of pancreas
7837.00	Open drainage of lesion of pancreas
7839z00	Open examination of pancreas NOS
7830.00	Transplantation of pancreas
7833600	Pancreatic necrosectomy
7836y00	Other specified other open operation on pancreatic duct
7838000	Division of annular pancreas
783A.00	Other open operations on pancreas
7831.00	Total excision of pancreas
7832.00	Excision of head of pancreas
7833400	Excision of tail of pancreas NEC
783A000	Open biopsy of lesion of pancreas
7836z00	Other open operation on pancreatic duct NOS
7838z00	Incision of pancreas NOS
7832000	Pancreaticoduodenectomy and excision of surrounding tissue
7837.11	Open drainage of cyst of pancreas
7839.00	Open examination of pancreas
783y.00	Other specified operations on pancreas
7832011	Childs pancreaticoduodenectomy
7830z00	Transplantation of pancreas NOS
7837300	Drainage of cyst of pancreas NEC
7837y00	Other specified open drainage of lesion of pancreas
7842y00	Other specified other operation on spleen
7840000	Total excision of spleen and replantation of fragments
7842500	Exploration of spleen
7841z00	Other excision of spleen NOS
784y.00	Other specified operations on spleen
7840.11	Total splenectomy
7842400	Banding of spleen
784..00	Spleen operations
7840400	Laparoscopic total splenectomy
7840y00	Other specified total excision of spleen
7840.00	Total excision of spleen
7841000	Partial splenectomy
7840z00	Total excision of spleen NOS
7840300	Splenectomy NEC
7842300	Repair of spleen
7842.00	Other operation on spleen
7842000	Transplantation of spleen
784z.00	Spleen operations NOS
7840100	Total splenectomy
7841.00	Other excision of spleen
7842z00	Other operation on spleen NOS
7841100	Marsupialisation of lesion of spleen

© 2021 Scott FI et al. *JAMA Network Open.*

7B26600	Detrusor myotomy
7B00y00	Other specified transplantation of kidney
7B12.14	Replantation of ureter
7B22.00	Enlargement or replacement of bladder
7B12z00	Reimplantation of ureter NOS
7B21.00	Partial excision of bladder
7B1..00	Ureter operations
7B10.00	Excision of ureter
7B10.11	Ureterectomy
7B2z.00	Bladder operations NOS
7B10200	Secondary ureterectomy
7B05111	Drainage of kidney NEC
7B20y00	Other specified total excision of bladder
7B02.00	Partial nephrectomy
7B06300	Exploration of renal transplant
7B15100	Intubated ureterotomy
7B11z00	Urinary diversion NOS
7B06.00	Other open operations on kidney
7B00400	Allotransplantation kidney from cadaver, heart non-beating
7B02y00	Other specified partial nephrectomy
7B15.00	Incision of ureter
7B04.00	Open repair of kidney
7B25.00	Open operations on contents of bladder
7B20.00	Total excision of bladder
7B05400	Therapeutic open aspiration of renal cyst
7B00.00	Transplantation of kidney
7B21y00	Other specified partial excision of bladder
7B22500	Caecal bladder augmentation
7B05100	Open nephrostomy or pyelostomy
7B03300	Rovsing's operation for polycystic kidney
7B03.11	Open removal of renal lesion
7B02300	Lower pole partial nephrectomy
7B12600	Intravesical unilateral ureteric reimplantation
7B11500	Ureterosigmoidostomy
7B01800	Nephroureterectomy with pluck lower ureterectomy
7B15200	Upper third ureterolithotomy
7B22900	Ileal bladder replacement
7B01600	Simple nephrectomy -live donor
7B12000	Unspecified bilateral reimplantation of ureter
7B22800	Clam ileocystoplasty
7B12611	Cohen unilateral reimplantation of ureter
7B21.11	Partial cystectomy
7B12.11	Bischoff replantation ureter
7B12500	Extravesical bilateral reimplantation of ureters
7B16y00	Other specified other open operation on ureter
7B02z00	Partial nephrectomy NOS
7B00200	Transplantation of kidney from cadaver
7B23400	Closure of exstrophy of bladder

© 2021 Scott FI et al. *JAMA Network Open.*

7B22700	Ileal augmentation of bladder
7B2D.00	Operations on bladder
7B11800	Creation of other continent conduit
7B11y00	Other specified urinary diversion
7B12.12	Hutch replantation of ureter
7B12200	Ureteric reimplantation after urinary diversion
7B16000	Open excision of ureterocele
7B26z00	Other open operation on bladder NOS
7B12300	Intravesical bilateral ureteric reimplantation
7B23000	Cystourethroplasty
7B0y.00	Other specified operations on kidney
7B12800	Extravesical unilateral reimplantation of ureter
7B05000	Unspecified open removal of calculus from kidney
7B01100	Nephroureterectomy-unspecified
7B2Dz00	Operations on bladder NOS
7B13200	Ileal replacement of ureter
7B13000	Anastomosis of ureter to bladder
7B01500	Transplant nephrectomy
7B0..11	Renal operations
7B12311	Cohen bilateral ureteric reimplantation
7B0..00	Kidney operations
7B22.11	Augmentation or replacement of bladder
7B15400	Lower third ureterolithotomy
7B25000	Open removal of bladder calculus
7B16100	Open excision of ureteric lesion
7B20100	Radical cystourethrectomy - female
7B26000	Open extirpation of bladder lesion
7B16300	Open biopsy of ureteric lesion
7B16600	Open exploration of ureter
7B04100	Revision of pyeloplasty
7B14.00	Repair of ureter
7B06y00	Other specified other open operation on kidney
7B20.11	Total cystectomy
7B20200	Radical cystoprostatectomy
7B02000	Heminephrectomy for duplex kidney
7B04400	Repair of renal laceration
7B20z00	Total excision of bladder NOS
7B04500	Plication and pyeloplasty of kidney
7B15z00	Incision of ureter NOS
7B04600	Anderson-Hynes pyeloplasty
7B00000	Autotransplant of kidney
7B03000	Open deroofing of renal cyst
7B00z00	Transplantation of kidney NOS
7B01200	Bilateral nephrectomy
7B04700	Culp pyeloplasty
7B05.00	Incision of kidney or renal pelvis
7B21z00	Partial excision of bladder NOS
7B10000	Total ureterectomy

© 2021 Scott FI et al. *JAMA Network Open.*

7B26500	Vesicostomy
7B02100	Division of isthmus of horseshoe kidney
7B01y00	Other specified total nephrectomy
7B12511	Leadbetter bilateral reimplantation of ureters
7B2y.00	Other specified operations on bladder
7B22z00	Enlargement of bladder NOS
7B22600	Caecal bladder replacement
7B26y00	Other specified other open operation on bladder
7B10y00	Other specified excision of ureter
7B23300	Repair of rupture of bladder
7B05z00	Incision of kidney NOS
7B06100	Open renal denervation
7B01000	Radical nephrectomy
7B20000	Radical cystoprostatourethrectomy
7B01011	Nephrectomy and excision of perirenal tissue
7B26300	Open biopsy of bladder lesion
7B01400	Simple nephrectomy - other
7B25100	Open removal of foreign body from bladder
7B04200	Nephropexy
7B25200	Cystostomy and removal of blood clot
7B20400	Cystectomy NEC
7B12512	Politano bilateral reimplantation of ureters
7B25z00	Open operation on contents of bladder NOS
7B11900	Creation of colonic conduit
7B16200	Ureterolysis
7B23z00	Other repair of bladder NOS
7B01.00	Total nephrectomy
7B11100	Unspecified other urinary intestinal diversion
7B05500	Therapeutic open aspiration of renal pelvis
7B04z00	Open repair of kidney NOS
7B12.00	Reimplantation of ureter
7B20300	Simple cystectomy
7B11011	Uretero-ileostomy, unspecified
7B2..00	Bladder operations
7B13.00	Other connection of ureter
7B0z.00	Kidney operations NOS
7B2Dy00	Other specified operations on bladder
7B11000	Construction of unspecified ileal conduit
7B12811	Leadbetter unilateral reimplantation of ureter
7B00111	Allotransplantation of kidney from live donor
7B03100	Open excision of renal lesion
7B10z00	Excision of ureter NOS
7B04.11	Pyeloplasty
7B13400	Revision of ureteric anastomosis
7B10300	Ureterectomy NEC
7B05y00	Other specified incision of kidney
7B1z.00	Ureter operations NOS
7B00100	Transplantation of kidney from live donor

© 2021 Scott FI et al. *JAMA Network Open.*

7B22000	Unspecified caecocystoplasty
7B01311	Excision of half of horseshoe kidney
7B11B00	Insertion of subcutaneous urinary diversion stent
7B11.00	Urinary diversion
7B16.00	Other open operations on ureter
7B21000	Diverticulectomy of bladder
7B04000	Other open pyeloplasty
7B22y00	Other specified enlargement of bladder
7B00211	Allotransplantation of kidney from cadaver
7B11600	Construction of standard ileal conduit
7B15111	Davis intubated ureterotomy
7B22B00	Colonic bladder replacement
7B20500	Cystourethrectomy
7B22300	Ileocaecocystoplasty
7B06000	Open renal biopsy
7B24.00	Open drainage of bladder
7B15.11	Ureterotomy
7B1y.00	Other specified operations on ureter
7B22100	Unspecified ileocystoplasty
7B11700	Construction of continent ileal conduit
7B23.00	Other repair of bladder
7B15000	Unspecified open ureterolithotomy
7B26200	Open transection of bladder
7B22.12	Enlargement of bladder
7B15y00	Other specified incision of ureter
7B24y00	Other specified open drainage of bladder
7B15300	Middle third ureterolithotomy
7B23y00	Other specified other repair of bladder
7B22200	Unspecified colocystoplasty
7B23100	Repair of vesicocolic fistula
7B01z00	Total nephrectomy NOS
7B13100	Boari flap anastomosis of ureter to bladder
7B22A00	Colonic bladder augmentation
7B16z00	Other open operation on ureter NOS
7B26.00	Other open operations on bladder
7B06200	Exploration of kidney
7B10100	Excision of segment of ureter
7B26400	Exploration of bladder
7B23500	Repair of fistula of bladder NEC
7B22400	Ileocaecal bladder replacement
7B06z00	Other open operation on kidney NOS
7B01.11	Total excision of kidney
7B00300	Allotransplantation of kidney from cadaver, heart-beating
7B02.11	Partial excision of kidney
7B12y00	Other specified reimplantation of ureter
7B04511	Hamilton plication and pyeloplasty of kidney
7B01700	Nephroureterectomy with open lower ureterectomy
7B02200	Upper pole partial nephrectomy

© 2021 Scott FI et al. *JAMA Network Open.*

7B01511	Excision of rejected transplanted kidney
7B01300	Heminephrectomy for horseshoe kidney
7B12100	Unspecified unilateral reimplantation of ureter
7B10400	Excision of duplex ureter
7B21100	Partial cystectomy
7B11200	Revision of urinary diversion
7E0y.00	Other specified operations on uterus
7E2..00	Ovary and broad ligament operations
7E06511	Hysterotomy NEC
7E04.12	Wertheim hysterectomy
7E2..12	Ovary operations
7E17z00	Open reversal of female sterilisation NOS
7E28200	Division of uteropelvic ligament
7E04711	Abdominal hysterectomy and left salpingoopherectomy
7E19011	Removal of ectopic pregnancy from fallopian tube
7E13.00	Partial excision of fallopian tube
7E19100	Drainage of fallopian tube
7E04E00	Laparoscopic subtotal hysterectomy
7E04312	Hysterectomy NEC
7E04D00	Excision of accessory uterus
7E04C00	Laparoscopic hysterectomy
7E04900	TAH - Tot abdom hysterectomy and BSO - bilat salpingophorect
7E21000	Open cauterisation of lesion of ovary
7E04600	Radical hysterectomy
7E18000	Reconstruction of fallopian tube
7E26011	Laparoscopic biopsy of ovary
7E16.00	Other open occlusion of fallopian tube
7E23000	Transposition of ovary
7E1B100	Open biopsy of fallopian tube
7E06112	Myomectomy
7E15011	Pomeroy open bilateral ligation of fallopian tubes
7E28.00	Operations on other uterine ligaments
7E23500	Oophorotomy and rupture of cyst
7E04300	Total abdominal hysterectomy NEC
7E27211	Open ventrosuspension
7E04A00	Abdominal hysterectomy with conservation of ovaries
7F1A000	Caesarean hysterectomy
7E16311	Open ringing of fallopian tube NEC
7E04511	Abdominal hysterectomy & bilateral salpingoophorectomy (BSO)
7E04.11	Abdominal hysterectomy
7E06711	Endometrial laser ablation
7E28011	Gilliam suspension of uterus
7E11z00	Unilateral excision of adnexa of uterus NOS
7E04500	Abdominal hysterectomy and bilateral salpingoophorectomy
7E26.11	Laparoscopy of ovary
7E06y00	Other specified open operation on uterus
7E28y00	Other specified operation on other uterine ligament
7E28400	Diathermy lesion uterosacral ligament

© 2021 Scott FI et al. *JAMA Network Open.*

7E28100	Plication of round ligament of uterus
7E18300	Suture of fallopian tube NEC
7E16000	Open ligation of remaining solitary fallopian tube
7E1B011	Salpingolysis - open
7E23300	Open drainage of cyst of ovary
7E06700	Endometrectomy
7E04700	Abdominal hysterectomy and right salpingoopherectomy
7E15100	Open bilateral clipping of fallopian tubes
7E1..00	Fallopian tube operations
7E11900	Left salpingectomy
7E11300	Salpingectomy of remaining solitary fallopian tube NEC
7E11600	Right salpingoophorectomy
7E17.11	Open reversal of tubal ligation
7E06600	Hysterotomy and termination of pregnancy
7E20000	Excision of wedge of ovary
7E20300	Ovarian cystectomy
7E06300	Open biopsy of lesion of uterus
7E16.12	Unilateral occlusion of fallopian tube
7E14200	Removal of tubal prosthesis from fallopian tube
7E15y00	Other specified open bilateral occlusion of fallopian tubes
7E04G00	Total abdominal hysterectomy with conservation of ovaries
7E28600	Suspension of uterus NEC
7E20200	Marsupialisation of lesion of ovary
7E13z00	Partial excision of fallopian tube NOS
7E13z11	Partial salpingectomy NEC
7E06000	Open removal of products of conception from uterus NEC
7E23y00	Other specified other open operation on ovary
7E22z00	Repair of ovary NOS
7E18200	Anastomosis of fallopian tube NEC
7E22200	Suture rupture corpus luteum
7E19.11	Salpingotomy
7E06z00	Open operation on uterus NOS
7E15.00	Open bilateral occlusion of fallopian tubes
7E16400	Open clipping of right fallopian tube
7E15111	Open bilateral ringing of fallopian tubes
7E20.11	Partial oophorectomy
7E23600	Oophorotomy
7E22y00	Other specified repair of ovary
7E04800	Abdominal hysterectomy and left salpingoophorectomy
7E20y00	Other specified partial excision of ovary
7E16100	Open ligation of fallopian tube NEC
7E18.00	Other repair of fallopian tube
7E11700	Left salpingoophorectomy
7E10000	Bilateral salpingoophorectomy
7E11500	Oophorectomy of remaining solitary ovary NEC
7E04311	Bonney abdominal hysterectomy
7E26000	Diagnostic endoscopic examination and biopsy lesion of ovary
7E17.00	Open reversal of female sterilisation

© 2021 Scott FI et al. *JAMA Network Open.*

7E19.00	Incision of fallopian tube
7E1C.12	Laparoscopic bilateral female sterilisation
7E17y00	Other specified open reversal of female sterilisation
7E1y.00	Other specified operations on fallopian tube
7E16300	Open clipping of fallopian tube NEC
7E18100	Replantation of fallopian tube
7E12.00	Other excision of adnexa of uterus
7E04000	Abdominal hysterocolpectomy and excision periuterine tissue
7E18y00	Other specified repair of fallopian tube
7E18z00	Repair of fallopian tube NOS
7E06500	Incision of uterus NEC
7E19y00	Other specified incision of fallopian tube
7E10100	Bilateral salpingectomy NEC
7E11400	Unilateral oophorectomy NEC
7E1A000	Excision of fimbria
7E1Ay00	Other specified operation on fimbria
7E19000	Removal of products of conception from fallopian tube
7E16700	Open ligation of left fallopian tube
7E15z00	Open bilateral occlusion of fallopian tubes NOS
7E22000	Replantation of ovary
7E13000	Excision of lesion of fallopian tube
7E21z00	Open destruction of lesion of ovary NOS
7E1A.00	Operations on fimbria
7E19012	Fimbrial extraction of tubal pregnancy
7E16600	Open ligation of right fallopian tube
7E1E.11	Laparoscopic reversal of female sterilisation
7E27200	Shortening of broad ligament of uterus
7E23z00	Other open operation on ovary NOS
7E27000	Excision of lesion of broad ligament of uterus
7E16z00	Other open occlusion of fallopian tube NOS
7E22300	Fixation of ovary NEC
7E06100	Open myomectomy
7E11000	Unilateral salpingoophorectomy NEC
7E21y00	Other specified open destruction of lesion of ovary
7E2..11	Broad ligament operations
7E04F00	Subtotal abdominal hysterectomy with conservation of ovaries
7E10.00	Bilateral excision of adnexa of uterus
7E0..00	Uterus operations
7E11.00	Unilateral excision of adnexa of uterus
7E18400	Salpingostomy
7E20100	Excision of lesion of ovary
7E1By00	Other specified open operation on fallopian tube
7E23.00	Other open operations on ovary
7E27z00	Operation on broad ligament of uterus NOS
7E04z00	Abdominal excision of uterus NOS
7E04200	Abdominal hysterocolpectomy NEC
7E27100	Destruction of lesion of broad ligament of uterus
7E27.00	Operations on broad ligament of uterus

© 2021 Scott FI et al. *JAMA Network Open.*

7E1B300	Exploration of fallopian tube
7E14000	Insertion of tubal prosthesis into fallopian tube
7E10200	Bilateral oophorectomy NEC
7E12z00	Other excision of adnexa of uterus NOS
7E21.00	Open destruction of lesion of ovary
7E04400	Subtotal abdominal hysterectomy
7E26z00	Diagnostic endoscopic examination of ovary NOS
7E04B00	Lapar total abdominal hysterect bilat salpingo-oophorectomy
7E20z00	Partial excision of ovary NOS
7E04y00	Other specified abdominal excision of uterus
7E11200	Unilateral salpingectomy NEC
7E15000	Open bilateral ligation of fallopian tubes
7E11800	Right salpingectomy
7E05600	Lap assist vag hysterectomy with bilat salpingo-oophorectomy
7E0z.00	Uterus operations NOS
7E25211	Laparoscopic drainage ovarian cyst
7E1A200	Excision of hydatid of Morgagni
7E15.11	Open bilateral female sterilisation
7E28z00	Operation on other uterine ligament NOS
7E1..11	Tubal operations - fallopian
7E16.11	Other open female sterilisation
7E06800	Repair of uterus
7E16y00	Other specified other open occlusion of fallopian tube
7E1z.00	Fallopian tube operations NOS
7E13100	Excision of ectopic ovarian pregnancy
7E28500	Suspension of uterus using mesh
7E28311	Laparoscopic ventrosuspension
7E26y00	Other specified diagnostic endoscopic examination of ovary
7E06011	Hysterotomy & evacuation retained products conception NEC
7E23200	Open biopsy of lesion of ovary
7E17100	Open removal of clip from fallopian tube NEC
7E06.00	Other open operations on uterus
7E12300	Right oophorectomy NEC
7E14100	Revision of tubal prosthesis in fallopian tube
7E06111	Excision fibroid
7E12000	Salpingoophorectomy NEC
7E06200	Open excision of lesion of uterus NEC
7E12400	Left oophorectomy NEC
7E1Az00	Operation on fimbria NOS
7E1Bz00	Open operation on fallopian tube NOS
7E19z00	Incision of fallopian tube NOS
7E22100	Suture of ovary
7E13300	Excision of ruptured ectopic tubal pregnancy
7E04100	Abdominal hysterectomy & excision of periuterine tissue NEC
7E13y00	Other specified partial excision of fallopian tube
7E28000	Suspension of uterus
7E17000	Reanastomosis of fallopian tube NEC
7E22.00	Repair of ovary

7E1A100	Burying of fimbria in wall of uterus
7E04512	TAH - total abdom hysterectomy & bilateral salpingoophorect
7E04.00	Abdominal excision of uterus
7E1B200	Open dilation of fallopian tube
7E11100	Salpingoophorectomy remaining solitary fallop tube and ovary
7E17.12	Open reversal of female sterilisation
7E23400	Oophorotomy and drainage of abscess
7E12100	Salpingectomy NEC
7E1B.00	Other open operations on fallopian tube
7E20.00	Partial excision of ovary
7E12200	Oophorectomy NEC
7A15300	Emerg bypass infrarenal aorta by anastom aorta to aorta NEC
7A34K00	Operation on aneurysm visceral branch of abdominal aorta NEC
7A1y.00	Other specified operations on aorta
7A12111	Aorto bifemoral graft
7A34G00	Open embolectomy of visceral branch of abdominal aorta NEC
7A13.11	Emergency repair of aortic aneurysm
7A33H00	Bypass of visceral branch of abdominal aorta NEC
7A15000	Emerg bypass ascending aorta by anastom aorta to aorta NEC
7A31z00	Other open operation on renal artery NOS
7A13400	Emerg replace aneurysm abdom aorta by anast aorta/aorta NEC
7A13300	Emerg replace aneurysm infrarenal aorta by anast aorta/aorta
7A34y00	Other open op on other visceral branch abdominal aorta OS
7A31y00	Other specified other open operation on renal artery
7A13000	Emerg replace aneurysm asc aorta by anastom aorta to aorta
7A34.13	Other open operations on superior mesenteric artery
7A10z00	Extraanatomic bypass of aorta NOS
7A30211	Replantation of renal artery
7A13y00	Emergency replacement of aneurysmal segment of aorta OS
7A19300	Operation on aortic body
7A12100	Bypass bifurc aorta by anastom aorta to femoral artery NEC
7A34000	Open embolectomy of coeliac artery NEC
7A31100	Ligation of renal artery
7A12y00	Other specified other bypass of bifurcation of aorta
7A11.00	Replacement of aneurysmal bifurcation of aorta
7A31300	Operation on aneurysm of renal artery
7A31.00	Other open operations on renal artery
7A14000	Replace aneurysm ascend aorta by anast of aorta/aorta NEC
7A34E00	Operation on aneurysm of inferior mesenteric artery NEC
7A34800	Open embolisation of coeliac artery NEC
7A34J00	Ligation of visceral branch of abdominal aorta NEC
7A12112	Dacron aortofemoral Y graft
7A30100	Bypass of renal artery
7A30000	Plastic repair and end to end anastomosis of renal artery
7A16y00	Other specified other bypass of segment of aorta
7A14.11	Aortic aneurysm repair
7A33.12	Reconstruction of inferior mesenteric artery
7A1z.00	Aorta operations NOS

© 2021 Scott FI et al. *JAMA Network Open.*

7A12300	Bypass bifurcation aorta by anastom aorta to iliac artery
7A11100	Replace aneurysm bifurc aorta by anast aorta to femoral art
7A14z00	Other replacement of aneurysmal segment of aorta NOS
7A16z00	Other bypass of segment of aorta NOS
7A19y00	Other specified other open operation on aorta
7A33900	Endarterectomy & patch repair superior mesenteric artery NEC
7A19200	Open embolectomy of bifurcation of aorta
7A34.00	Other open operations other visceral branch abdominal aorta
7A10300	Axillo-unifemoral PTFE bypass graft
7A34.12	Other open operations on inferior mesenteric artery
7A10100	Bypass aorta by anastomosis axillary to femoral artery NEC
7A13411	Tube graft abdominal Aortic aneurysm (emergency)
7A12z00	Other bypass of bifurcation of aorta NOS
7A12312	Dacron aortoiliac Y graft
7A14300	Replace aneurys infrarenal aorta by anast aorta to aorta NEC
7A30y00	Other specified reconstruction of renal artery
7A16000	Bypass of ascending aorta by anastomosis aorta to aorta NEC
7A15.00	Other emergency bypass of segment of aorta
7A10y00	Other specified extraanatomic bypass of aorta
7A12311	Aorto biiliac graft
7A19.00	Other open operations on aorta
7A1..11	Dacron graft operations on aorta
7A18300	Release of vascular ring of aorta
7A34A00	Open embolisation of inferior mesenteric artery NEC
7A11311	Y graft abdominal Aortic aneurysm
7A33y00	Reconstruction of other visceral branch abdominal aorta OS
7A16400	Bypass of abdominal aorta by anastomosis aorta to aorta NEC
7A34100	Open embolectomy of superior mesenteric artery NEC
7A18500	Plastic repair of aorta and insertion of tube graft
7A18.00	Plastic repair of aorta
7A34D00	Operation on aneurysm of superior mesenteric artery NEC
7A18600	Repair of interrupted aortic arch
7A18100	Plastic repair of aorta using subclavian flap
7A17000	Revision of prosthesis of thoracic aorta
7A19z00	Other open operation on aorta NOS
7A14200	Replace aneurys suprarenal aorta by anast aorta to aorta NEC
7A33.11	Reconstruction of coeliac artery
7A11200	Emerg repl aneurysm bifurc aorta by anast aorta to iliac a
7A18000	Plastic repair of aorta and end to end anastomosis of aorta
7A11300	Replace aneurysm bifurc aorta by anast aorta to iliac artery
7A14411	Tube graft of Abdominal aortic aneurysm
7A17z00	Attention to prosthesis of aorta NOS
7A18z00	Plastic repair of aorta NOS
7A11z00	Replacement of aneurysmal bifurcation of aorta NOS
7A18y00	Other specified plastic repair of aorta
7A1..00	Aorta operations
7A31200	Open embolisation of renal artery
7A33.13	Reconstruction of superior mesenteric artery

© 2021 Scott FI et al. *JAMA Network Open.*

7A3..00	Branches of abdominal aorta operations
7A33000	Bypass of coeliac artery NEC
7A14y00	Other replacement of aneurysmal segment of aorta OS
7A19400	Operation on aneurysm of aorta NEC
7A18200	Plastic repair of aorta using patch graft
7A17200	Revision of prosthesis of abdominal aorta NEC
7A14400	Replace aneurysm abdominal aorta by anast aorta to aorta NEC
7A30.00	Reconstruction of renal artery
7A33100	Bypass of superior mesenteric artery NEC
7A11211	Y graft of abdominal Aortic aneurysm (emergency)
7A30400	Translocation of branch of renal artery
7A14.00	Other replacement of aneurysmal segment of aorta
7A16.00	Other bypass of segment of aorta
7A34600	Ligation of inferior mesenteric artery NEC
7A31000	Open embolectomy of renal artery
7A30z00	Reconstruction of renal artery NOS
7A14100	Replace aneurysm thoracic aorta by anast of aorta/aorta NEC
7A13100	Emerg replace aneurysm thor aorta by anastom aorta to aorta
7A10200	Axillo-bifemoral bypass graft
7A12000	Emerg bypass bifurc aorta by anast aorta to femoral artery
7A34.11	Other open operations on coeliac artery
7A33.00	Reconstruction of other visceral branch of abdominal aorta
7A12.00	Other bypass of bifurcation of aorta
7A13.00	Emergency replacement of aneurysmal segment of aorta
7A10.00	Extraanatomic bypass of aorta
7A30200	Reimplantation of renal artery
7A16300	Bypass of infrarenal aorta by anastomosis aorta to aorta NEC
7A18111	Hamilton repair coarctation of aorta using subclavian flap
7A17100	Revision of prosthesis of bifurcation of aorta
7A13z00	Emergency replacement of aneurysmal segment of aorta NOS
7H28z00	Therapeutic endoscopic operation on peritoneum NOS
7H23100	Excision of lesion of omentum
7H23z00	Operation on omentum NOS
7H24000	Excision of lesion of mesentery of small intestine
7H23400	Creation of omental flap
7H24.00	Operations on mesentery of small intestine
7H23.00	Operations on omentum
7H24z00	Operation on mesentery of small intestine NOS
7H22y00	Other specified opening of peritoneal cavity
7H29400	Diagnostic endo ultras exam perit biop intraabdominal organ
7H28y00	Therapeutic endoscopic operation on peritoneum OS
7H23511	Morison omentopexy
7H26200	Biopsy of lesion of posterior peritoneum
7H25000	Excision of lesion of mesentery of colon
7H22400	Open instillation therapeutic substance in abdominal cavity
7H26z00	Operation on posterior peritoneum NOS
7H22000	Exploratory laparotomy
7H29z00	Diagnostic endoscopic examination of peritoneum NOS

© 2021 Scott FI et al. *JAMA Network Open.*

7H2C500	Biopsy of abdominal mass
7H28300	Endoscopic removal of foreign body from peritoneum
7H28000	Endoscopic resection of lesion of peritoneum
7H28.00	Therapeutic endoscopic operations on peritoneum
7H22700	Reopen abdomen and reexplore intraabdominal op site NEC
7H26.00	Operations on posterior peritoneum
7H29100	Diag endoscopy of peritoneum & biopsy intraabdom organ NEC
7H28100	Endoscopic destruction of lesion of peritoneum
7H29.13	Diagnostic laparoscopy
7H28.11	Therapeutic laparoscopic operations on peritoneal cavity
7H22600	Reopen abdo reexplore intraabd op site surg arr postop bleed
7H22100	Laparotomy and removal of foreign body from abdominal cavity
7H23300	Biopsy of lesion of omentum
7H23y00	Other specified operation on omentum
7H25z00	Operation on mesentery of colon NOS
7H22z00	Opening of peritoneal cavity NOS
7H2z.00	Peritoneum operations NOS
7H22112	Removal of pack from peritoneal cavity
7H22300	Reopening of laparotomy site
7H29.00	Diagnostic endoscopic examination of peritoneum
7H25200	Biopsy of lesion of mesentery of colon
7H2Cz00	Other operation on peritoneum NOS
7H2y.00	Other specified operations on peritoneum
7H29200	Diagnostic laparoscopy of female pelvis
7H23500	Omentopexy
7H25.00	Operations on mesentery of colon
7H25300	Repair of mesentery of colon
7H27000	Open biopsy of lesion of peritoneum NEC
7H2C600	Biopsy of pelvic mass
7H29y00	Diagnostic endoscopic examination of peritoneum OS
7H29211	Gynaecological laparoscopy NEC
7H23000	Omentectomy
7H29000	Diagnostic endoscopic exam and biopsy lesion of peritoneum
7H22.11	Opening of abdomen
7H24y00	Other specified operation on mesentery of small intestine
7H29300	Diagnostic endoscopic ultrasound examination of peritoneum
7H22011	Laparotomy
7H29011	Laparoscopic biopsy peritoneum
7H29.11	Diagnostic laparoscopic examination of peritoneal cavity
7H24200	Biopsy of lesion of mesentery of small intestine
7H22111	Removal of pack from abdominal cavity
7H22.00	Opening of peritoneal cavity
7H22200	Opening of abdomen and exploration of groin
7H26000	Excision of lesion of posterior peritoneum
7H27.00	Other open operations on peritoneum
7H29.12	Peritoneoscopy

Diagnostic codes were identified within the READ code system representing procedures within the abdomen. A total of 1435 codes were identified.

© 2021 Scott FI et al. *JAMA Network Open.*

eTable 2. Diagnostic Codes used to identify surgical events in Optum Clinformatics

ICD 9 Surgical Codes and Their Description	
44.38	Laparoscopic gastroenterostomy
44.67	Laparoscopic procedures for creation of esophagogastric sphincteric competence
44.68	Laparoscopic gastroplasty
44.95	Laparoscopic gastric restrictive procedure
44.96	Laparoscopic revision of gastric restrictive procedure
44.97	Laparoscopic removal of gastric restrictive device(s)
44.98	(Laparoscopic) adjustment of size of adjustable gastric restrictive device
45.81	Laparoscopic total intra-abdominal colectomy
47.01	Laparoscopic appendectomy
47.11	Laparoscopic incidental appendectomy
48.51	Laparoscopic abdominoperineal resection of the rectum
50.14	Laparoscopic liver biopsy
50.25	Laparoscopic ablation of liver lesion or tissue
51.23	Laparoscopic cholecystectomy
51.24	Laparoscopic partial cholecystectomy
53.42	Laparoscopic repair of umbilical hernia with graft or prosthesis
53.43	Other laparoscopic umbilical herniorrhaphy
53.62	Laparoscopic incisional hernia repair with graft or prosthesis
53.63	Other laparoscopic repair of other hernia of anterior abdominal wall with graft or prosthesis
53.71	Laparoscopic repair of diaphragmatic hernia, abdominal approach
53.83	Laparoscopic repair of diaphragmatic hernia, with thoracic approach
54.11	Exploratory laparotomy
54.12	Reopening of recent laparotomy site
54.19	Other laparotomy
54.21	Laparoscopy
54.51	Laparoscopic lysis of peritoneal adhesions
65.01	Laparoscopic oophorotomy
65.13	Laparoscopic biopsy of ovary
65.14	Other laparoscopic diagnostic procedures on ovaries
65.23	Laparoscopic marsupialization of ovarian cyst
65.24	Laparoscopic wedge resection of ovary
65.25	Other laparoscopic local excision or destruction of ovary
65.31	Laparoscopic unilateral oophorectomy
65.41	Laparoscopic unilateral salpingo-oophorectomy
65.53	Laparoscopic removal of both ovaries at same operative episode
65.54	Laparoscopic removal of remaining ovary
65.63	Laparoscopic removal of both ovaries and tubes at same operative episode
65.64	Laparoscopic removal of remaining ovary and tube
65.74	Laparoscopic simple suture of ovary
65.75	Laparoscopic reimplantation of ovary
65.76	Laparoscopic salpingo-oophoroplasty
65.81	Laparoscopic lysis of adhesions of ovary and fallopian tube
68.31	Laparoscopic supracervical hysterectomy [LSH]
68.41	Laparoscopic total abdominal hysterectomy
68.61	Laparoscopic radical abdominal hysterectomy
42.42	Esophagectomy
42.51	Intrathoracic esophagoesophagostomy
42.52	Intrathoracic esophagogastrostomy

© 2021 Scott FI et al. *JAMA Network Open.*

42.53	Intrathoracic esophageal anastomosis with interposition of small bowel
42.54	Other intrathoracic esophagoenterostomy
42.55	Intrathoracic esophageal anastomosis with interposition of colon
42.56	Other intrathoracic esophagocolostomy
42.58	Intrathoracic esophageal anastomosis with other interposition
42.59	Other intrathoracic anastomosis of esophagus
42.61	Antesternal esophagoesophagostomy
42.62	Antesternal esophagogastrostomy
42.63	Antesternal esophageal anastomosis with interposition of small bowel
42.64	Other antesternal esophagoenterostomy
42.65	Antesternal esophageal anastomosis with interposition of colon
42.66	Other antesternal esophagocolostomy
42.68	Other antesternal esophageal anastomosis with interposition
42.69	Other antesternal anastomosis of esophagus
43.0	Gastrotomy
43.11	Percutaneous [endoscopic] gastrostomy [PEG]
43.19	Other gastrostomy
43.3	Pyloromyotomy
43.41	Endoscopic excision or destruction of lesion or tissue of stomach
43.42	Local excision of other lesion or tissue of stomach
43.49	Other destruction of lesion or tissue of stomach
43.5	Partial gastrectomy with anastomosis to esophagus
43.6	Partial gastrectomy with anastomosis to duodenum
43.7	Partial gastrectomy with anastomosis to jejunum
43.81	Partial gastrectomy with jejunal transposition
43.89	Other partial gastrectomy
43.91	Total gastrectomy with intestinal interposition
43.99	Other total gastrectomy
44.00	Vagotomy, not otherwise specified
44.01	Truncal vagotomy
44.02	Highly selective vagotomy
44.03	Other selective vagotomy
44.11	Transabdominal gastroscopy
44.15	Open biopsy of stomach
44.31	High gastric bypass
44.32	Percutaneous [endoscopic] gastrojejunostomy
44.39	Other gastroenterostomy without gastrectomy
44.40	Suture of peptic ulcer, not otherwise specified
44.41	Suture of gastric ulcer site
44.42	Suture of duodenal ulcer site
44.5	Revision of gastric anastomosis
44.61	Suture of laceration of stomach
44.62	Closure of gastrostomy
44.63	Closure of other gastric fistula
44.64	Gastropexy
44.65	Esophagogastroplasty
44.69	Other repair of stomach
44.99	Other operations on stomach
45.00	Incision of intestine, not otherwise specified
45.01	Incision of duodenum
45.02	Other incision of small intestine
45.03	Incision of large intestine

45.11	Transabdominal endoscopy of small intestine
45.15	Open biopsy of small intestine
45.16	Esophagogastroduodenoscopy [EGD] with closed biopsy
45.19	Other diagnostic procedures on small intestine
45.21	Transabdominal endoscopy of large intestine
45.26	Open biopsy of large intestine
45.27	Intestinal biopsy, site unspecified
45.32	Other destruction of lesion of duodenum
45.34	Other destruction of lesion of small intestine, except duodenum
45.41	Excision of lesion or tissue of large intestine
45.49	Other destruction of lesion of large intestine
45.50	Isolation of intestinal segment, not otherwise specified
45.51	Isolation of segment of small intestine
45.52	Isolation of segment of large intestine
45.61	Multiple segmental resection of small intestine
45.62	Other partial resection of small intestine
45.63	Total removal of small intestine
45.71	Open and other multiple segmental resection of large intestine
45.72	Open and other cecectomy
45.73	Open and other right hemicolectomy
45.74	Open and other resection of transverse colon
45.75	Open and other left hemicolectomy
45.76	Open and other sigmoidectomy
45.79	Other and unspecified partial excision of large intestine
45.82	Open total intra-abdominal colectomy
45.83	Other and unspecified total intra-abdominal colectomy
45.90	Intestinal anastomosis, not otherwise specified
45.91	Small-to-small intestinal anastomosis
45.92	Anastomosis of small intestine to rectal stump
45.93	Other small-to-large intestinal anastomosis
45.94	Large-to-large intestinal anastomosis
45.95	Anastomosis to anus
46.01	Exteriorization of small intestine
46.02	Resection of exteriorized segment of small intestine
46.03	Exteriorization of large intestine
46.04	Resection of exteriorized segment of large intestine
46.10	Colostomy, not otherwise specified
46.11	Temporary colostomy
46.13	Permanent colostomy
46.14	Delayed opening of colostomy
46.20	Ileostomy, not otherwise specified
46.21	Temporary ileostomy
46.22	Continent ileostomy
46.23	Other permanent ileostomy
46.24	Delayed opening of ileostomy
46.31	Delayed opening of other enterostomy
46.32	Percutaneous (endoscopic) jejunostomy [PEJ]
46.39	Other enterostomy
46.40	Revision of intestinal stoma, not otherwise specified
46.41	Revision of stoma of small intestine
46.42	Repair of pericolostomy hernia
46.43	Other revision of stoma of large intestine

46.50	Closure of intestinal stoma, not otherwise specified
46.51	Closure of stoma of small intestine
46.52	Closure of stoma of large intestine
46.60	Fixation of intestine, not otherwise specified
46.61	Fixation of small intestine to abdominal wall
46.62	Other fixation of small intestine
46.63	Fixation of large intestine to abdominal wall
46.64	Other fixation of large intestine
46.71	Suture of laceration of duodenum
46.72	Closure of fistula of duodenum
46.73	Suture of laceration of small intestine, except duodenum
46.74	Closure of fistula of small intestine, except duodenum
46.75	Suture of laceration of large intestine
46.76	Closure of fistula of large intestine
46.79	Other repair of intestine
46.80	Intra-abdominal manipulation of intestine, not otherwise specified
46.81	Intra-abdominal manipulation of small intestine
46.82	Intra-abdominal manipulation of large intestine
46.85	Dilation of intestine
46.93	Revision of anastomosis of small intestine
46.94	Revision of anastomosis of large intestine
46.97	Transplant of intestine
46.99	Other operations on intestines
47.09	Other appendectomy
47.19	Other incidental appendectomy
47.2	Drainage of appendiceal abscess
47.91	Appendicostomy
47.92	Closure of appendiceal fistula
47.99	Other operations on appendix
48.0	Proctotomy
48.1	Proctostomy
48.21	Transabdominal proctosigmoidoscopy
48.43	Open pull-through resection of rectum
48.49	Other pull-through resection of rectum
48.50	Abdominoperineal resection of the rectum, not otherwise specified
48.52	Open abdominoperineal resection of the rectum
48.59	Other abdominoperineal resection of the rectum
48.61	Transsacral rectosigmoidectomy
48.62	Anterior resection of rectum with synchronous colostomy
48.63	Other anterior resection of rectum
48.64	Posterior resection of rectum
48.65	Duhamel resection of rectum
48.69	Other resection of rectum
48.71	Suture of laceration of rectum
48.72	Closure of proctostomy
48.73	Closure of other rectal fistula
48.74	Rectorectostomy
48.75	Abdominal proctopexy
48.76	Other proctopexy
48.79	Other repair of rectum
48.81	Incision of perirectal tissue
48.82	Excision of perirectal tissue

© 2021 Scott FI et al. *JAMA Network Open.*

48.91	Incision of rectal stricture
48.92	Anorectal myectomy
48.93	Repair of perirectal fistula
48.99	Other operations on rectum and perirectal tissue
50.12	Open biopsy of liver
50.21	Marsupialization of lesion of liver
50.22	Partial hepatectomy
50.23	Open ablation of liver lesion or tissue
50.24	Percutaneous ablation of liver lesion or tissue
50.26	Other and unspecified ablation of liver lesion or tissue
50.29	Other destruction of lesion of liver
50.3	Lobectomy of liver
50.4	Total hepatectomy
50.51	Auxiliary liver transplant
50.59	Other transplant of liver
50.61	Closure of laceration of liver
50.69	Other repair of liver
51.02	Trocar cholecystostomy
51.03	Other cholecystostomy
51.04	Other cholecystotomy
51.13	Open biopsy of gallbladder or bile ducts
51.21	Other partial cholecystectomy
51.22	Cholecystectomy
51.31	Anastomosis of gallbladder to hepatic ducts
51.32	Anastomosis of gallbladder to intestine
51.33	Anastomosis of gallbladder to pancreas
51.34	Anastomosis of gallbladder to stomach
51.35	Other gallbladder anastomosis
51.36	Choledochoenterostomy
51.37	Anastomosis of hepatic duct to gastrointestinal tract
51.39	Other bile duct anastomosis
51.41	Common duct exploration for removal of calculus
51.42	Common duct exploration for relief of other obstruction
51.51	Exploration of common duct
51.61	Excision of cystic duct remnant
51.62	Excision of ampulla of Vater (with reimplantation of common duct)
51.63	Other excision of common duct
51.91	Repair of laceration of gallbladder
51.92	Closure of cholecystostomy
51.93	Closure of other biliary fistula
51.94	Revision of anastomosis of biliary tract
51.99	Other operations on biliary tract
52.12	Open biopsy of pancreas
52.51	Proximal pancreatectomy
52.52	Distal pancreatectomy
52.53	Radical subtotal pancreatectomy
52.59	Other partial pancreatectomy
52.6	Total pancreatectomy
52.7	Radical pancreaticoduodenectomy
52.80	Pancreatic transplant, not otherwise specified
52.81	Reimplantation of pancreatic tissue
52.82	Homotransplant of pancreas

52.83	Heterotransplant of pancreas
52.95	Other repair of pancreas
52.96	Anastomosis of pancreas
52.99	Other operations on pancreas
53.00	Unilateral repair of inguinal hernia, not otherwise specified
53.01	Other and open repair of direct inguinal hernia
53.02	Other and open repair of indirect inguinal hernia
53.03	Other and open repair of direct inguinal hernia with graft or prosthesis
53.04	Other and open repair of indirect inguinal hernia with graft or prosthesis
53.05	Repair of inguinal hernia with graft or prosthesis, not otherwise specified
53.10	Bilateral repair of inguinal hernia, not otherwise specified
53.11	Other and open bilateral repair of direct inguinal hernia
53.12	Other and open bilateral repair of indirect inguinal hernia
53.13	Other and open bilateral repair of inguinal hernia, one direct and one indirect
53.14	Other and open bilateral repair of direct inguinal hernia with graft or prosthesis
53.15	Other and open bilateral repair of indirect inguinal hernia with graft or prosthesis
53.16	Other and open bilateral repair of inguinal hernia, one direct and one indirect, with graft or prosthesis
53.17	Bilateral inguinal hernia repair with graft or prosthesis, not otherwise specified
53.41	Other and open repair of umbilical hernia with graft or prosthesis
53.49	Other open umbilical herniorrhaphy
53.51	Incisional hernia repair
53.59	Repair of other hernia of anterior abdominal wall
53.61	Other open incisional hernia repair with graft or prosthesis
53.69	Other and open repair of other hernia of anterior abdominal wall with graft or prosthesis
53.72	Other and open repair of diaphragmatic hernia, abdominal approach
53.75	Repair of diaphragmatic hernia, abdominal approach, not otherwise specified
53.80	Repair of diaphragmatic hernia with thoracic approach, not otherwise specified
53.81	Plication of the diaphragm
53.82	Repair of parasternal hernia
53.84	Other and open repair of diaphragmatic hernia, with thoracic approach
53.9	Other hernia repair
54.0	Incision of abdominal wall
54.23	Biopsy of peritoneum
54.3	Excision or destruction of lesion or tissue of abdominal wall or umbilicus
54.4	Excision or destruction of peritoneal tissue
54.59	Other lysis of peritoneal adhesions
54.61	Reclosure of postoperative disruption of abdominal wall
54.62	Delayed closure of granulating abdominal wound
54.63	Other suture of abdominal wall
54.64	Suture of peritoneum
54.71	Repair of gastroschisis
54.72	Other repair of abdominal wall
54.73	Other repair of peritoneum
54.74	Other repair of omentum
54.75	Other repair of mesentery
54.92	Removal of foreign body from peritoneal cavity
54.95	Incision of peritoneum
54.96	Injection of air into peritoneal cavity
65.09	Other oophorotomy
65.19	Other diagnostic procedures on ovaries
65.21	Marsupialization of ovarian cyst
65.22	Wedge resection of ovary

© 2021 Scott FI et al. *JAMA Network Open.*

65.29	Other local excision or destruction of ovary
65.39	Other unilateral oophorectomy
65.49	Other unilateral salpingo-oophorectomy
65.51	Other removal of both ovaries at same operative episode
65.52	Other removal of remaining ovary
65.61	Other removal of both ovaries and tubes at same operative episode
65.62	Other removal of remaining ovary and tube
65.71	Other simple suture of ovary
65.72	Other reimplantation of ovary
65.73	Other salpingo-oophoroplasty
65.79	Other repair of ovary
65.89	Other lysis of adhesions of ovary and fallopian tube
65.91	Aspiration of ovary
65.92	Transplantation of ovary
65.93	Manual rupture of ovarian cyst
65.94	Ovarian denervation
65.95	Release of torsion of ovary
65.99	Other operations on ovary
66.01	Salpingotomy
66.02	Salpingostomy
66.11	Biopsy of fallopian tube
66.19	Other diagnostic procedures on fallopian tubes
68.13	Open biopsy of uterus
68.14	Open biopsy of uterine ligaments
68.15	Closed biopsy of uterine ligaments
68.16	Closed biopsy of uterus
68.39	Other and unspecified subtotal abdominal hysterectomy
68.49	Other and unspecified total abdominal hysterectomy
68.69	Other and unspecified radical abdominal hysterectomy
68.9	Other and unspecified hysterectomy
69.29	Other repair of uterus and supporting structures
70.72	Repair of colovaginal fistula
70.73	Repair of rectovaginal fistula
17.11	Laparoscopic repair of direct inguinal hernia with graft or prosthesis
17.12	Laparoscopic repair of indirect inguinal hernia with graft or prosthesis
17.13	Laparoscopic repair of inguinal hernia with graft or prosthesis, not otherwise specified
17.21	Laparoscopic bilateral repair of direct inguinal hernia with graft or prosthesis
17.22	Laparoscopic bilateral repair of indirect inguinal hernia with graft or prosthesis
17.23	Laparoscopic bilateral repair of one direct and one indirect inguinal hernia with graft or prosthesis
17.24	Laparoscopic bilateral repair of inguinal hernia with graft or prosthesis, not otherwise specified
17.31	Laparoscopic multiple segmental resection of large intestine
17.32	Laparoscopic cecectomy
17.33	Laparoscopic-assisted right colectomy
17.34	Laparoscopic resection of transverse colon
17.35	Laparoscopic left hemicolectomy
17.36	Laparoscopic sigmoidectomy
17.39	Other laparoscopic partial excision of large intestine

CPT Surgical Codes and Their Description

43644	Surgical laparoscopy with gastric bypass and Roux-en-Y gastroenterostomy
1007372	Laparoscopic Procedures on the Stomach
43842	Gastric restrictive procedure, without gastric bypass, for morbid obesity; other than vertical-banded gastroplasty
43774	Laparoscopy, surgical, gastric restrictive procedure; removal of adjustable gastric restrictive device and subcutaneous port components

© 2021 Scott FI et al. *JAMA Network Open.*

43771	Laparoscopy, surgical, gastric restrictive procedure; removal of adjustable gastric restrictive device and subcutaneous port components
44212	Laparoscopy, surgical; colectomy, total, abdominal, with proctectomy, with ileostomy
44970	Laparoscopic appendectomy
44970	Laparoscopic appendectomy
1007664	Excisional Laparoscopic Procedures on the Rectum
49321	Surgical laparoscopy with biopsy
1007835	Laparoscopy, surgical, ablation of 1 or more liver tumor(s)
47562	Cholecystectomy, Laparoscopic
47562	Cholecystectomy, Laparoscopic
49652	Laparoscopy, surgical, repair, ventral, umbilical, spigelian or epigastric hernia (includes mesh insertion, when performed); reducible
49659	Surgical laparoscopy with herniorrhaphy
49654	Laparoscopy, surgical, repair, incisional hernia (includes mesh insertion, when performed); reducible
49540	Repair of abdominal muscle herniation
39503	Repair of diaphragmatic hernia
49000	Exploratory laparotomy
49002	Reopening of recent laparotomy
1007953	Incision Procedures on the Abdomen
58660	Laparoscopic lysis of adhesions
58679	Laparoscopy procedure on ovary
49321	Surgical laparoscopy with biopsy
58679	Laparoscopy procedure on ovary
58661	Surgical laparoscopy with partial oophorectomy
58720	Unilateral partial/complete salpingo-oophorectomy
1008681	Gynecologic Surgical Procedures
58679	Laparoscopy procedure on ovary
58541	Surgical laparoscopy with supracervical hysterectomy
58570	Surgical hysteroscopy with complete hysterectomy
1013911	Hysterectomy
1007214	Surgical Procedure on the Esophagus
43320	Esophagogastrostomy by transthoracic approach
43320	Esophagogastrostomy by transabdominal approach
1007346	Gastrotomy
43520	Cutting of pyloric muscle
43601	Excision of Lesion of Stomach
43620	Gastrectomy, total with esophagoenterostomy
43631	Gastrectomy, partial, distal; with gastroduodenostomy
43632	Gastrectomy, partial, distal; with gastrojejunostomy
1007363	Gastrectomy, partial, distal
1007359	Gastrectomy, total
43640	Vagotomy, Truncal
43640	Vagotomy, Truncal
43640	Selective vagotomy
1014146	Gastric restrictive procedure, with gastric bypass for morbid obesity
43820	Gastrojejunostomy
43501	suture of bleeding stomach ulcer
43840	suture of duodenal injury
1007410	Revision of gastroduodenal anastomosis with reconstruction
43840	suture of gastric injury
43870	Closure of gastrostomy
43999	Operation on stomach
1007423	Incision Procedure on Intestines

© 2021 Scott FI et al. *JAMA Network Open.*

1006964	Digestive System Surgical Procedures
1007427	Enterotomy, small intestine, other than duodenum
1007423	Incision Procedure on Intestines
44799	Procedure on small intestine
45399	Large bowel procedure
1007422	Surgical Procedures on the Intestines
44799	Procedure on small intestine
44110	enterotomy and excision of lesion of large intestine
1007433	Excision Procedures on the Intestines
44130	Anastomosis of the Intestine
44799	Procedure on small intestine
44320	Colostomy Procedure
44310	Ileostomy or jejunostomy, non-tube
44300	Open placement of enterostomy tube
44372	small intestinal endoscopy; with placement of percutaneous jejunostomy tube
1007496	Enterostomy- external fistulization of intestines procedure
1007545	Repair procedures on the intestines (except rectum)
44346	Repair of paracolstomy hernia
1007545	Repair procedures on the intestines (except rectum)
44620	Closure of stoma of small intestine
1007422	Surgical Procedures on the Intestines
43840	Suture of duodenal wound
1007546	Suture of small intestine for perforated ulcer, diverticulum, wound, injury or rupture
44799	Procedure on small intestine
44604	Suture of large intestine for perforated ulcer, diverticulum, wound, injury or rupture
1007545	Repair procedures on the intestines (except rectum)
1007422	Surgical Procedures on the Intestines
44130	Anastomosis of small intestine
44136	Transplantation of donor small bowel
1007652	Other operations on intestines
44950	Appendectomy
44955	Appendectomy; when done for indicated purpose at time of other major procedure (not as separate procedure) (List separately in addition to code for primary procedure)
44900	Incision and drainage of appendiceal abscess, open
45399	Large bowel procedure
1007578	Operation on appendix
1007592	Incision of rectum
45999	Procedure on rectum
45111	Proctectomy; partial resection of rectum, transabdominal approach
45135	Repair of prolapsed rectum
1007591	Surgical Procedures on the Colon and Rectum
45540	Repair of rectal prolapse by abdominal approach
1007671	Repair of rectum
1007596	Excision Procedures on the Rectum
1007795	Surgical Procedures on the Liver
47300	Marsupialization of cyst of abscess of liver
47120	Partial hepatectomy
1007840	Ablation, open, of 1 or more liver tumor(s)
4783	Ablation, 1 or more liver tumor(s), percutaneous, cryoablation
1007840	Ablation, open, of 1 or more liver tumor(s)
1007795	Surgical Procedures on the Liver
1007806	Hepatectomy, resection of liver
1007811	Transplantation of liver

© 2021 Scott FI et al. *JAMA Network Open.*

1007845	Biliary Tract surgical Procedures
1007827	Repair of liver
47600	Cholecystectomy procedure
47612	Cholecystectomy with exploration of common duct with choledochoenterostomy
1007878	Biliary tract excision
48148	Exicison of ampulla of Vater
1007892	Biliary Tract repair
47600	Cholecystectomy procedure
48100	Open biopsy of pancreas
1007914	Pancreatectomy
48140	Distal subtotal pancreatectomy
48999	Pancreas Procedure
1007944	Pancreas Transplantation
1007934	Repair of pancreas
48999	Procedures on Pancreas
49521	Repair of incarcerated or strangulated recurrent inguinal hernia
1008001	Hernioplasty, Herniorrhaphy, Herniotomy Procedures
49560	Repair of incisional or abdominal hernia
39503	Repair of diaphragmatic hernia
1007953	Abdomen, Peritoneum and Omentum: Incision
49999	Procedure on abdomen, peritoneum, and omentum
1007971	Excision and Destruction Procedures on the Abdomen, Peritoneum and Omentum
1008054	Suture Procedures on the Abdomen, Peritoneum, and Omentum
49605	Repair of gastroschisis
100800	Abdomen, Peritoneum and Omentum: Repair
1007570	Surgical Procedues on Meckel's Diverticulum and Mesentery
49402	Removal of foreign body from peritoneal cavity
1007953	Abdomen, Peritoneum and Omentum: incision
49400	Injection of air or contrast into peritoneal cavity
1008915	Incision of ovary
1008914	Operation on Ovary
58925	Removal of ovarian cysts
58920	Wedge resection of ovary
58940	Unilateral oophorectomy
58720	Salpingo-oophorectomy, complete or partial, unilateral or bilateral (separate procedure
58970	Aspiration of eggs from ovaries
1008919	drainage of ovarian abscess
1008890	Incision Procedures on the Oviduct/Ovary
558770	Salpingostomy
1008681	Surgical Procedures on the Female Genital System
58100	Endometrial sampling (biopsy) with or without endocervical sampling (biopsy), without cervical dilation, any method (separate procedure)
59525	This is for partial hysterectomy
58150	Complete abdominal hysterectomy
1013911	Hysterectomy
1008681	Surgical Procedures on the Female Genital System
59300	Reapir of vagina
59300	Reapir of vagina
49650	Surgical laparoscopy with repair of inguinal hernia
44238	Laparoscopy procedure on intestine
1007479	Laparoscopic excision procedures on the intestines

© 2021 Scott FI et al. *JAMA Network Open.*

eTable 3. Baseline characteristics of the study cohort within THIN stratified by the presence of adhesion-related complications

Variable	No adhesion-related complication	Any adhesion-related complication		Small bowel obstruction*	
		N	Unadjusted incidence rate (per 100 person years, 95% CI)	N	Unadjusted Incidence rate
	146,541	2,060 (1.39%)	0.29 (0.28, 0.31)	1,489 (1.0%)	0.21 (0.20, 0.22)
Sex					
Male	43,645 (29.8%)	805 (39.1%)	0.42 (0.40, 0.46)	722 (48,5%)	0.38 (0.35, 0.41)
Female	102, 896 (70.2%)	1255 (60.9%)	0.24 (0.23, 0.26)	767 (51.5%)	0.15 (0.14, 0.16)
Age					
18-44	66,613 (45.5%)	612 (29.7%)	0.18 (0.17, 0.20)	199 (13.4%)	0.06 (0.05, 0.07)
45-64	46,211 (31.5%)	642 (31.2%)	0.28 (0.26, 0.30)	535 (35.9%)	0.23 (0.21, 0.25)
65+	33,717 (23.0%)	806 (39.1%)	0.60 (0.56, 0.64)	755 (50.7%)	0.56 (0.52, 0.60)
Medical comorbidities prior to surgery					
Diabetes	11,783 (8.0%)	152 (7.4%)	0.35 (0.30, 0.41)	134 (9%)	0.31 (0.26, 0.37)
Obesity	40,997 (28.0%)	437 (21.2%)	0.27 (0.22, 0.32)	318 (21.4%)	0.19 (0.16, 0.23)
Hyperlipidemia	6,840 (4.7%)	111 (5.4%)	0.48 (0.30, 0.77)	93 (6.3%)	0.36 (0.21, 0.63)
Hypertension	22,856 (15.6%)	385 (18.7%)	0.40 (0.36, 0.44)	341 (22.9%)	0.36 (0.32, 0.40)
Tobacco use	87,918 (60%)	1,315 (63.8%)	0.33 (0.31, 0.34)	960 (64.5%)	0.24 (0.22, 0.25)
Cancer history	16,342 (11.2%)	508 (24.7%)	0.86 (0.79, 0.94)	463 (31.1%)	0.79 (0.72, 0.86)
Surgical site					
Bowel Surgery	32,453 (22.2%)	857 (41.6%)	0.61 (0.57, 0.65)	754 (50.6%)	0.54 (0.50, 0.58)
Non-bowel surgery	114,088 (77.9%)	1203 (58.4%)	0.21 (0.57, 0.65)	735 (49.4%)	0.13 (0.12, 0.14)
Medication use at the time of surgery					
Statin use	17,096 (11.7%)	281 (13.6%)	0.43 (0.38, 0.49)	251 (16.9%)	0.39 (0.34, 0.44)
Fibrate use	856 (0.6%)	14 (0.7%)	0.47 (0.28, 0.79)	13 (0.9%)	0.43 (0.25, 0.75)
Former Statin use	3,428 (2.3%)	51 (2.5%)	0.74 (0.56, 0.97)	46 (3.1%)	0.66 (0.50, 0.89)
ACE/ARB use	18,722 (12.6%)	312 (15.2%)	0.42 (0.38, 0.47)	278 (18.7%)	0.37 (0.33, 0.42)

*Small bowel obstructions were a subgroup of any adhesion-related complications.

© 2021 Scott FI et al. *JAMA Network Open.*

eTable 4. Association between statin use and covariates with small bowel obstruction in THIN

Exposure	Completely adjusted model (HR, 95% CI)	Parsimonious model* (HR, 95% CI)
Statin use at the time of surgery	0.83 (0.71, 0.96)	0.80 (0.70, 0.92)
Female gender	0.85 (0.76, 0.95)	
Age (reference: 18-44)		
45-64	3.70 (3.13, 4.37)	3.71 (3.15, 4.38)
65+	6.29 (5.29, 7.47)	6.27 (5.30, 7.42)
Hypertension	0.91 (0.80, 1.04)	
Diabetes	0.88 (0.73, 1.06)	
Hyperlipidemia	1.05 (0.60, 1.81)	
Obesity	0.93 (0.75, 1.15)	
Tobacco use	1.20 (1.08, 1.34)	1.21 (1.09, 1.35)
Surgical site involving the bowel	2.75 (2.46, 3.08)	2.86 (2.57, 3.18)
History of malignancy	1.72 (1.52, 1.94)	1.74 (1.54, 1.96)

*Parsimonious model created through stepwise backwards elimination, with remaining covariates

© 2021 Scott FI et al. *JAMA Network Open.*

eTable 5. Baseline characteristics of surgical cohort within Optum, stratified by the presence of adhesion related complications

Variable	No adhesion-related complication	Any adhesion-related complication		Small bowel obstruction*	
		N	Unadjusted incidence rate	N	Unadjusted Incidence rate
	1,134,081	54,136 (4.6%)	1.36 (1.35,1.37)	29,364 (2.5%)	0.72 (0.72,0.73)
Sex					
Male	310,849 (27.4%)	14,690 (27.1%)	1.36 (1.34, 1.38)	11,507 (39.2%)	1.06 (1.04, 1.08)
Female	823,232 (72.6%)	39,446 (72.9%)	1.36 (1.34, 1.37)	17,857 (60.8%)	0.60 (0.59, 0.61)
Age					
18-44	448,295 (39.5%)	18,459 (34.1%)	1.22 (1.21, 1.24)	5,915 (20.1%)	0.38 (0.37, 0.39)
45-64	507,649 (44.8%)	21,955 (40.6%)	1.20 (1.19, 1.22)	12,372 (42.1%)	0.67 (0.66, 0.68)
65+	178,137 (15.7%)	13,722 (25.3%)	2.09 (2.06, 2.13)	11,077 (37.7%)	1.67 (1.64, 1.70)
Medical comorbidities prior to surgery					
Hypertension	306,237 (27.0%)	19,275 (35.6%)	1.78 (1.76, 1.81)	12,589 (42.9%)	1.15 (1.13, 1.17)
Diabetes	163,090 (14.4%)	10,610 (19.6%)	1.94 (1.90, 1.97)	6,886 (23.5%)	1.23 (1.20, 1.26)
Obesity	158,977 (14.0%)	9,692 (17.9%)	1.88 (1.84, 1.91)	5,261 (17.9%)	1.00 (0.97, 1.02)
Hyperlipidemia	470,581 (41.5%)	25,418 (47.0%)	1.53 (1.51, 1.54)	15,899 (54.1%)	0.94 (0.92, 0.95)
Tobacco use	100,630 (8.9%)	6,164 (11.4%)	1.94 (1.89, 1.99)	3,843 (13.1%)	1.19 (1.15, 1.23)
Cancer history	115,800 (10.2%)	10,935 (20.2%)	2.69 (2.64, 2.74)	8,142 (27.7%)	1.97 (1.92, 2.01)
Surgical site					
Bowel Surgery	156,748 (13.8%)	12,773 (23.6%)	2.41 (2.37, 2.45)	9,560 (32.6%)	1.78 (1.74, 1.81)
Non-bowel surgery	977,333 (86.2%)	41,363 (76.4%)	1.120 (1.18, 1.21)	19,804 (67.4%)	0.56 (0.56, 0.57)
Medication use at the time of surgery					
Statin use	121,504 (10.7%)	6,722 (12.4%)	1.59 (1.55, 1.62)	4,498 (15.3%)	1.05 (1.01, 1.08)
Fibrate use	4,322 (0.4%)	236 (0.4%)	1.67 (1.47, 1.90)	164 (0.6%)	1.15 (0.99, 1.34)
Former Statin use	95,207 (8.4%)	5,580 (10.3%)	1.71 (1.66, 1.75)	3,811 (13.0%)	1.15 (1.11, 1.19)

Baseline characteristics for those in Optum without an outcome of interest and those with adhesion-related complication (ARC) or small bowel obstruction (SBO). Incidence rates presented per 100 person years. *SBOs are a subgroup of ARCs.

© 2021 Scott FI et al. *JAMA Network Open.*

eTable 6. Association between statin use and covariates with small bowel obstruction in Optum

Exposure	Fully Adjusted model* (HR, 95% CI)
Statin use at the time of surgery	0.88 (0.85, 0.91)
Male gender	1.16 (1.13, 1.19)
Age (reference: 18-44)	Ref
45-64	1.63 (1.58, 1.68)
65+	3.08 (2.97, 3.19)
Hypertension	1.23 (1.19, 1.26)
Diabetes	1.16 (1.12, 1.19)
Hyperlipidemia	0.93 (0.91, 0.96)
Obesity	1.34 (1.29, 1.38)
Tobacco use	1.41 (1.37, 1.46)
Surgical site involving the bowel	2.51 (2.45, 2.58)
History of malignancy	1.99 (1.94, 2.05)

*Backwards elimination was not performed given findings of fully adjusted model in Optum

© 2021 Scott FI et al. *JAMA Network Open.*

eTable 7. Multivariable model assessing the association between former statin use, fibrate use, and adhesion-related complications (ARCs) and small bowel obstruction (SBO) after surgery in Optum's Clinformatics® Data Mart

Exposure	Parsimonious model in Optum*	
	Any adhesion-related complication (HR, 95% CI)	Small bowel obstruction (HR, 95% CI)
Never statin use	Ref	Ref
Former statin use	0.99 (0.96, 1.02)	1.01 (0.97, 1.05)
Statin use at the time of surgery	0.92 (0.89, 0.95)	0.89 (0.85, 0.92)
Fibrate use at the time of surgery	0.96 (0.84, 1.09)	0.96 (0.82, 1.12)
Male Gender	0.79 (0.77, 0.80)	1.16 (1.13, 1.19)
Age: 18-44	Ref	Ref
45-64	0.94 (0.93, 0.96)	1.63 (1.58, 1.68)
65+	1.37 (1.34, 1.41)	3.07 (2.96, 3.19)
Diabetes	1.17 (1.14, 1.20)	1.16 (1.12, 1.19)
Obesity	1.28 (1.25, 1.31)	1.34 (1.29, 1.38)
Hyperlipidemia	0.96 (0.94, 0.98)	0.93 (0.91, 0.96)
Hypertension	1.21 (1.19, 1.24)	1.23 (1.19, 1.26)
Tobacco use	1.31 (1.27, 1.34)	1.41 (1.37, 1.46)
History of malignancy	1.82 (1.78, 1.86)	1.99 (1.94, 2.05)
Surgical site involving the bowel	1.87 (1.83, 1.91)	2.51 (2.45, 2.58)

*Parsimonious model developed via backwards elimination in primary analyses. Multivariable model from primary analysis was used with the addition of variables for former statin use and fibrate use to assess their association with the outcome and effect on concurrent statin use.

© 2021 Scott FI et al. *JAMA Network Open.*

eTable 8. Multivariable model assessing the association between statin dose intensity and adhesion-related complications (ARCs) and small bowel obstruction (SBO) after surgery in The Health Improvement Network

Exposure	Adhesion-related complications (HR, 95%CI	Small Bowel Obstruction (HR, 95% CI)
Statin use at the time of surgery		
Low intensity	0.95 (0.68, 1.32)	0.94 (0.66, 1.33)
Moderate Intensity	0.78 (0.67, 0.91)	0.79 (0.67, 0.93)
High Intensity	0.77 (0.52, 1.14)	0.69 (0.44, 1.07)
Age (reference: 18-44)		
45-64	1.45 (1.29, 1.62)	3.72 (3.15, 4.38)
65+	2.27 (2.01, 2.56)	6.26 (5.29, 7.41)
Tobacco use	1.21 (1.11, 1.33)	1.21 (1.09, 1.35)
Surgical site involving the bowel	2.17 (1.98, 2.38)	2.86 (2.57, 3.19)
History of malignancy	1.81 (1.61, 2.02)	1.74 (1.54, 1.96)

*Parsimonious model developed via backwards elimination in primary analyses. Multivariable model from primary analysis was used with the addition of statin use stratified by LDL lowering intensity.

© 2021 Scott FI et al. *JAMA Network Open.*

eTable 9. Multivariable model assessing the association between statin use at the time of surgery and adhesion-related complications (ARCs) and small bowel obstruction (SBO) after surgery in The Health Improvement Network, adjusting for year of initial surgery and country of origin

Exposure	Adhesion-related complications (HR, 95%CI)	Small Bowel Obstruction (HR, 95% CI)
Statin use at the time of surgery	0.84 (0.73, 0.96)	0.86 (0.74, 0.99)
Age (reference: 18-44)		
45-64	1.45 (1.29, 1.62)	3.71 (3.14, 4.37)
65+	2.25 (1.99, 2.53)	6.16 (5.21, 7.29)
Tobacco use	1.21 (1.11, 1.33)	1.23 (1.11, 1.38)
Surgical site involving the bowel	2.17 (1.98, 2.38)	2.86 (2.57, 3.18)
History of malignancy	1.82 (1.63, 2.05)	1.78 (1.57, 2.01)
Year of incident surgery	0.98 (0.97, 0.99)	0.96 (0.95. 0.98)
Country (reference: England)		
Wales	0.81 (0.67, 0.98)	0.74 (0.58, 0.94)
Scotland	1.10 (0.95, 1.29)	1.17 (0.98, 1.40)
Northern Ireland	0.92 (0.72, 1.17)	0.88 (0.66, 1.18)

*Parsimonious model developed via backwards elimination in primary analyses. Multivariable model from primary analysis was used with the addition of surgical year, included as a continuous variable including all years within THIN, and country of origin. The cohort of interest within THIN includes data from England (81.1%), Wales (6.4%), Scotland (8.8%), and Northern Ireland (3.7%).

© 2021 Scott FI et al. *JAMA Network Open.*

eTable 10. Association between statin use at the time of surgery and ARCs and SBOs occurring within 5, 4, 3, 2, and 1 years of the incident surgical event

	Required window for outcome to have occurred					
Number of events in each time window	Any time during follow-up	5 years	4 years	3 years	2 years	1 year
ARCs	2,060	1,764	1,638	1,494	1,255	874
SBOs	1,489	1,270	1,184	1,078	916	645
HR of statins	HR (95% CI)	HR (95% CI)	HR (95% CI)	HR (95% CI)	HR (95% CI)	HR (95% CI)
ARCs		0.79 (0.69, 0.91)	0.80 (0.70, 0.93)	0.80 (0.69, 0.93)	0.81 (0.68, 0.95)	0.82 (0.68, 0.998)
SBOs		0.79 (0.68, 0.91)	0.79 (0.68, 0.93)	0.79 (0.68, 0.93)	0.80 (0.68, 0.96)	0.85 (0.96. 1.03)

Association between statin use at the time of surgery and ARCs and SBOs occurring at any time during follow-up, as well as only considering events that occurred within 5, 4, 3, 2, or 1 years of the incident surgical event. HRs presented here were calculated in parsimonious adjusted model.

© 2021 Scott FI et al. *JAMA Network Open.*

eFigure. Covariate balance with 1:1 matching in propensity score analyses

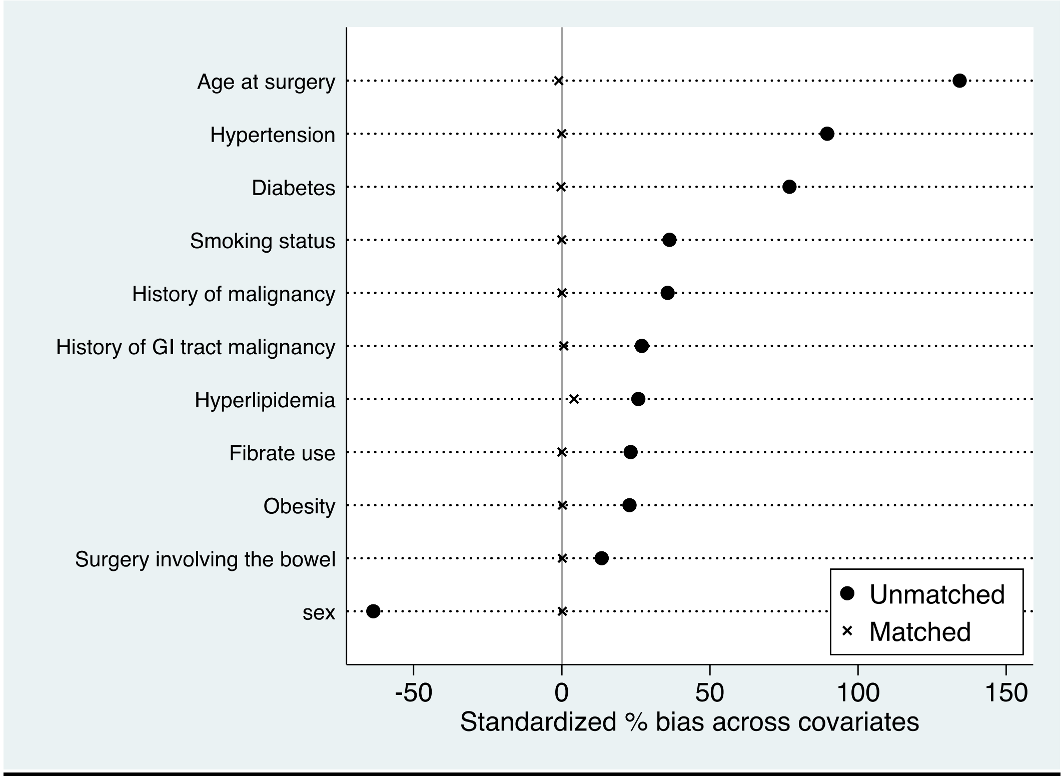

Covariate balance between statin exposed and unexposed individuals at the time of surgery, prior to and after propensity score matching.

© 2021 Scott FI et al. *JAMA Network Open.*

eReferences

1. Leuven E, Sianesi B. PSMATCH2: Stata module to perform full Mahalanobis and propensity score matching, common support graphing, and covariate imbalance testing. Boston College Department of Economics. https://ideas.repec.org/c/boc/bocode/s432001.html. Published 2016. Accessed2020.
2. Garrido MM, Kelley AS, Paris J, et al. Methods for Constructing and Assessing Propensity Scores. *Health Services Research.* 2014;49(5):1701-1720.
3. Vajravelu RK, Osterman MT, Aberra FN, et al. Indeterminate QuantiFERON-TB Gold Increases Likelihood of Inflammatory Bowel Disease Treatment Delay and Hospitalization. *Inflamm Bowel Dis.* 2017;24(1):217-226.
4. VanderWeele TJ, Ding P. Sensitivity Analysis in Observational Research: Introducing the E-Value. *Ann Intern Med.* 2017;167(4):268-274.

© 2021 Scott FI et al. *JAMA Network Open.*
